# Comparing Images of Depression in Mass Media and AI-Generated Pictures: Mixed Methods Study

**DOI:** 10.2196/81230

**Published:** 2026-04-14

**Authors:** Nuria Saladie, Carolina Llorente, Renée Joosten, Gema Revuelta

**Affiliations:** 1 Science, Communication and Society Studies Centre, Department of Medicine and Life Sciences, Universitat Pompeu Fabra, Carrer Doctor Aiguader 88, Barcelona, 08003, Spain, 34 933160912

**Keywords:** mental health, depression, artificial intelligence, mass media, mental health communication

## Abstract

**Background:**

Images play an important role in reducing stigma related to mental health, which often is distorted in the media. In recent years, generative artificial intelligence (AI) has been used to generate images related to mental health. However, first reports suggest that AI-generated images do not depict mental health conditions accurately. In-depth studies on the topic of mental health representations in AI-generated images are still missing.

**Objective:**

The main objective of this study is to analyze and compare the visual representation of depression in mass media and in AI-generated images.

**Methods:**

The methodologies used were discussion groups (15 participants) and a quasi-experimental online survey (792 interviewees), aimed at people with depression and young people.

**Results:**

The results showed that both the images used in the media and those generated by AI reproduced stereotypes and stigmas about depression. However, participants considered AI-generated pictures to be more stereotypical, stigmatizing, and more likely to have a negative impact on people with depression. In contrast, media images were considered more appropriate, realistic, inclusive, and that better reflected the relationship between gender and depression. Statistically significant differences were observed between the control and test groups in both people with depression and young people (*P*<.001), indicating that when people were aware of what images were AI-generated, they tended to reject them to a greater extent.

**Conclusions:**

Considering the current trend toward the widespread use of AI in mental health communication, it is crucial to promote closer collaborations between science journalists, AI developers, and mental health experts, including patients’ associations, as well as a shift toward user-participatory AI design.

## Introduction

### Background

The worldwide depression incidence increased during 2020 due to the COVID-19 pandemic, although before then, depression already featured as a leading burden of disease globally [1]. According to the World Health Organization, approximately 280 million people have depression worldwide, the equivalent of 5% of the adult population [2]. Young people are among the most affected groups [3-5], with depression rates having doubled from prepandemic estimates [6]. Spain was one of the countries in Europe most affected by COVID-19 infections, complications, and deaths, with young people being among the groups (together with frontline health care workers) with higher prevalence rates of mental health symptoms [7]. The prevalence of depression in Spain increased from 3.06% prepandemic to 12% postpandemic [8]. At an informational level, Google searches for mental health disorders have tripled for some pathologies compared with 2016, which highlights that society needs, and is actively searching for, information on mental health [9].

Mental health stigma and discrimination have been described as having worse consequences than the mental health conditions themselves do [10]. Higher levels of self-stigma in people with mental health issues result in lower treatment adherence [11], which is related to the fact that internalized stigma and treatment-related stigma are negatively associated with seeking help [12]. In Spain, the stigmatization of mental health conditions is prevalent in stereotypes, prejudices, and discrimination associated with people with mental health issues, as shown by a recent systematic review [13].

When studying mental health stigmatization, it is necessary to pay attention to the images that accompany the text, especially since multiple studies conclude that negative media representations of mental health lead to mental health stigma [14-16]. In light of this, framing images positively or negatively can significantly affect the way audiences react to messages [17]. Positive visual information can reduce stigma related to mental health treatments; improve information, understanding, and effectiveness in identifying symptoms; promote empowerment; and foster better communication between patients, family members, and medical professionals [18,19].

Against this background, it is evident that the media play a key role: they can contribute not only to the creation and perpetuation of stereotypes and prejudices but also to the reduction of stigma surrounding mental health by portraying realistic, balanced, or even positive counterstereotypical examples [20]. In reality, however, mental health coverage by the media is insufficient and often resorts to stigmatizing archive images depicting hopelessness [9]. An analysis of a wide range of media revealed that descriptions of mental illness are often distorted by misinformation, exaggeration, and inaccuracies [14]. Similarly, the media, including news and social media, were found to display mental health issues in a negative light, although an increasing number of balancing stories appeared, mainly in magazines [15]. Images of depression on the social media platform Tumblr mostly depicted crying women, were often very dark-colored, and represented gloominess and sadness [21]. On Instagram, images of public self-disclosure of mental health issues express emotional distress, calls for help, and displays of vulnerability [22].

In this context, artificial intelligence (AI) could become a mental health ally. Studies have reported potential benefits of integrating AI in health communication, which include improving access to health information, supporting health behavior change, addressing diverse community needs [23], or boosting the reach and effectiveness of public health communication strategies [24]. Particularly for mental health, studies show that AI presents opportunities to enhance mental health care, for example, by serving as a complementary tool to bridge gaps in conventional mental health services, especially in areas with limited resources or high social stigma [25], developing algorithms to analyze images and texts shared on social media to diagnose or predict depression [26], or supporting the mental health of health professionals with AI chatbots [27]. However, AI use in mental health also presents some ethical challenges [25,28], as it can make systematic errors in its decision-making processes leading to unfair outcomes, also called bias [29]. First reports suggest that AI-generated images do not depict mental health conditions accurately [30,31] and that they frequently reflect cultural stereotypes and historical visual tropes, including gender biases and stigma [32]. Nonetheless, in-depth studies on the topic of mental health representations in AI-generated images are still missing.

From a theoretical perspective, the social impact of media and AI-generated images can be explained through complementary approaches from media effects research, social psychology, and technology acceptance studies. Framing theory posits that visual elements, such as color, lighting, posture, and scene context, shape how mental health is interpreted, guiding audience attributions of realism, stigma, vulnerability, or social support [17,33,34]. However, media effects are not uniform: reception studies emphasize that individuals actively interpret images through their personal experiences, beliefs, emotional states, and social identities, which may modulate responses across age, gender, and lived experience with depression [35,36]. From a psychosocial perspective, social representations and emotional anchoring processes contribute to the stabilization of shared visual stereotypes of depression in collective memory [37,38]. Finally, differential evaluations of media versus AI-generated images can be interpreted through the technology acceptance model (TAM), which proposes that perceived usefulness and perceived ease of use shape behavioral intentions toward technology [39] and that these perceptions may vary across demographic groups, such as gender and age, due to differences in prior technological experience [40], as well as attitudinal biases toward AI-generated products [41,42].

### Study Objectives

The main objective of this study is to analyze and compare the visual representation of depression in mass media and in AI-generated images. To do so, we explore the perceptions of 2 strategic population groups: people with depression and young people. The specific research questions (RQs) are as follows:

- RQ1. How is the representation of depression in media and AI-generated images perceived?
- RQ2. How do the opinions of people with depression differ from the opinions of young people regarding the depiction of depression in media and AI-generated images?
- RQ3. How do people with depression recommend better illustrating depression, and how do these recommendations compare to those by young people?
- RQ4. How do people with depression, as well as young people, receive recommendations from mental health associations regarding how to illustrate depression?

## Methods

Two types of methods were implemented: one qualitative (discussion groups) and one quantitative (survey). A mixed methods approach was used because of the greater depth and breadth of information that it can offer compared to using 1 type of method alone [43].

### Discussion Groups

In total, 3 discussion groups were organized involving 15 people (13 women and 2 men): one was with people with depression and two with young people. The distribution of the discussion groups organized is available in Multimedia Appendix 1.

Each discussion group had a tailored script approved by the Advisory Board of the project (formed by a mixed group of professionals from the fields of clinical psychology, science journalism, and representatives from mental health associations). A slide show was prepared for discussion groups containing 64 images, of which 30 were found in the media, another 30 were AI-generated, and 4 were related to recommendations from mental health associations.

Media images were obtained from 3 different Spanish newspapers by providing their inbuilt search engine with the keyword “depression” (in Spanish, “depresión”) on December 18, 2023. The 10 most recent images were selected for each newspaper, spanning from December 15, 2023, to January 4, 2019. The 3 newspapers selected were *La Razón*, *El País*, and *Eldiario.es*. These newspapers represent different ideologies, from more conservative to more left-leaning ideologies, and traditional and online-native media [44]. Out of the 30 images from the media, 22 included women, 7 included men, and 1 was an ungendered illustration. In 6 images, the figure of the person was silhouetted against the light. Eleven images were of celebrities. On 1 occasion, 2 people interacted with each other, while all other pictures contained either a single person or people not interacting. In 2 instances, images depicted a woman holding a baby. Only 1 image was in black and white, and the rest were in color. These images are available in Multimedia Appendix 2.

AI-generated images were obtained from 4 different tools in June 2023: Deep Dream Generator (Stable Diffusion 1.5), DeepAI (Stable Diffusion 1.5), OpenAI (DALL·E 2), and Dream Studio (Stable Diffusion 2.1). Images were created using the prompt “depression.” Standard parameters were applied to generate the images, with no particular filters or prompt settings. Out of the 30 AI-generated images, 10 depicted women, 11 depicted men, and 9 were unknown or ungendered. The most repeated type of image was that of a person sitting down, either with their hands to their head or face, or holding their knees with their arms, looking down (18 images). Eleven pictures were in black and white, and 19 were in color. In addition, 3 were illustrations, and 27 represented realistic pictures. These images are available in Multimedia Appendix 3.

Images recommended by mental health associations were obtained from the guides from the Confederación Salud Mental España, “Style guide on mental health for the media” [45], and from Cochrane, “Choosing images for sharing evidence: a guide” [46].

Discussion groups took place between the 11th and 18th of January 2024. Conversations were registered and later transcribed. The coding and qualitative analysis were carried out via the qualitative research support program ATLAS.ti (version 22).

### Ethical Considerations

Before commencing work, the research project was evaluated by a panel of independent experts of the Spanish Foundation for Science and Technology (FECYT), who deemed the data management protocol as adequate. Following this evaluation, a bioethics committee and an ethical protocol were not considered necessary because no high-risk interventions were involved. Before conducting the discussion groups, informed consent was obtained from all participants, who provided it voluntarily and with the full knowledge of the facts.

All appropriate measures have been taken to comply with applicable regulations on the protection of personal data, including the General Data Protection Regulation and the Spanish Organic Law 3/2018 of December 5 on the Protection of Personal Data and Guarantee of Digital Rights [47]. At Universitat Pompeu Fabra, the Institutional Commission for the Ethical Review of Projects was responsible for the ethical assessment and monitoring of research involving human participants, human biological samples, or personal data (including health and clinical data). As this study did not involve any of these elements, ethical review and approval were not required according to institutional and national regulations.

### Survey

Based on the qualitative analysis of the discussion groups, a quasi-experimental survey was developed. The insights contributed by discussion groups’ participants offered contextual depth and nuance regarding lived experiences and perspectives. Specific topics that appeared in the discussion groups and that were later transferred to the survey included, among others, how images could be more inclusive, better illustrate the relationship between gender and depression, or could negatively affect people with depression. Based on the most commonly voiced opinions regarding how the images could be improved, a list of options was prepared for the survey asking respondents to select the phrases that could help improve the way depression was illustrated.

The survey consisted of 14 questions and was validated twice before being shared: first, by the Advisory Board of the project; and second, by discussion group participants. The survey is available in Multimedia Appendix 4.

The survey had 2 versions: a test and a control (a mechanism specific to quasi-experimental surveys). In the test version, respondents knew which images were from the media and which ones were AI-generated; in the control version, respondents were not told the difference.

Target respondents were people with depression and young people. The first 7 questions of the survey asked respondents to select up to 5 images out of the 30 presented (15 from the media and 15 AI-generated). The remaining 7 questions were either multiple choice, scale, or open text questions.

The survey was sent to target respondents via an online opt-in panel. The sample was representative of Spanish society in terms of population distribution, encompassing all autonomous communities, age groups, genders, education levels, and population sizes. The data collection period was from the 22nd to 24th of May 2024. A total of 792 responses were obtained—390 from people with depression and 402 from young people. These numbers surpassed the minimum required to obtain a statistical robustness of 95% with an error margin of 5% (achieved at 385 responses for each target group).

Regarding the screening of participants, the first question of the survey asked them whether a doctor had diagnosed them with any mental health condition, and a list of 6 options was offered, including depression. Participants who ticked that option were labeled under “People with depression.” People from this group were not restricted by their age because their defining trait for the purposes of the study was the fact that they had been diagnosed with depression. On the other hand, people who had not been diagnosed with any mental health issues and were 18 to 29 years old were labeled under “Young people.”

Raw data were cleaned in Excel. For statistical analysis, all data were imported into the R software (R Core Team). The data were initially tested for normality, and the corresponding tests were run (Pearson chi-square test, Fisher exact test, logistic regressions, Mann-Whitney *U* test, and Kruskal-Wallis test, specified in text). For all analyses, a statistical threshold of *α*=.05 was used. Violin plots were used to better represent the full distribution of nonparametric data.

## Results

The results are presented following the study’s research questions.

### How Is the Representation of Depression in Media and AI-Generated Images Perceived?

Both media and AI-generated images reproduce stereotypes and stigma about depression. However, respondents perceive AI-generated images more negatively than media images. In particular, AI-generated images are considered to be even more stereotypical, reproducing stigmas of marginalization or social exclusion and negatively affecting people with depression. In contrast, media images are considered relatively more appropriate, realistic, inclusive, and that represent the relationship between gender and depression better.

### How Do the Opinions of People With Depression Differ From the Opinions of Young People Regarding the Depiction of Depression in Media and AI-Generated Images?

To answer this question, we present the results in 2 categories, depending on whether the questions were framed (1) positively or (2) negatively.

#### Responses to Positively Framed Questions

The questions that were framed positively asked which images were considered more appropriate, realistic, inclusive, and better represented the relationship between gender and depression.

A nonparametric Pearson chi-square analysis was carried out to examine differences between the test and control groups among people with depression. There were statistically significant differences between the 2 groups (*P*<.001), with the test group (ie, people who knew which images were from the media and which were AI-generated) choosing media images more frequently (2374/3652, 65.01% chose media; 1278/3652, 34.99% chose AI images). On the other hand, the control group (those who did not know whether images were from the media or AI) selected a higher percentage of AI-generated images (1590/3650, 43.56% chose media; 2060/3650, 56.44% chose AI images).

**Figure 1.**
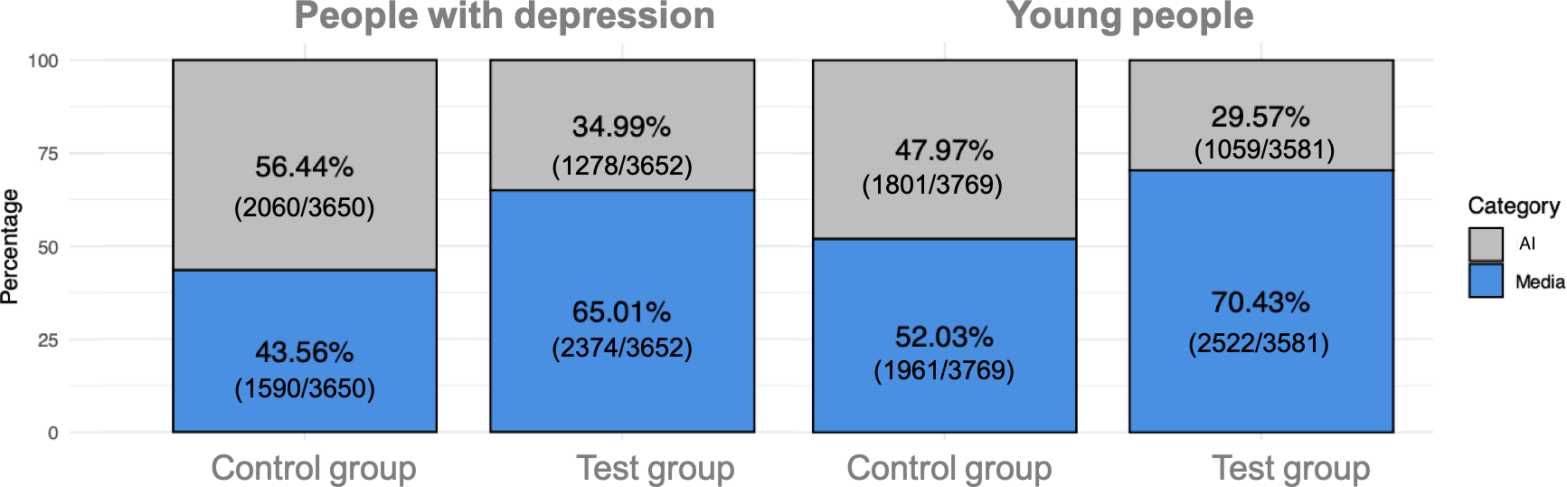
Distribution of answers for positively framed questions regarding media or artificial intelligence (AI)–generated images, segregated by group among people with depression and young people.

The same trend was observed in responses from young people (*P*<.001)—while the test group chose media images more frequently (2522/3581, 70.43% chose media; 1059/3581, 29.57% chose AI), the ratio in the control group was much more balanced (1961/3769, 52.03% chose media; 1801/3769, 47.97% chose AI; Figure 1).

The top 3 images selected for positively framed questions among people with depression were all from the media for the test group, whereas the control group included 2 out of 3 that were AI-generated. Among young people, the same was observed: while the test group chose only media images in the top 3 positions, the control group had a more balanced selection. Among people with depression, the first images selected both by the test and control groups represented women sitting down, hiding their face in their arms. Among young people, both the test and control groups selected in the first place the same image, showing a woman holding a baby. Images are available as MultimediaAppendices 2  3.

**Figure 2.**
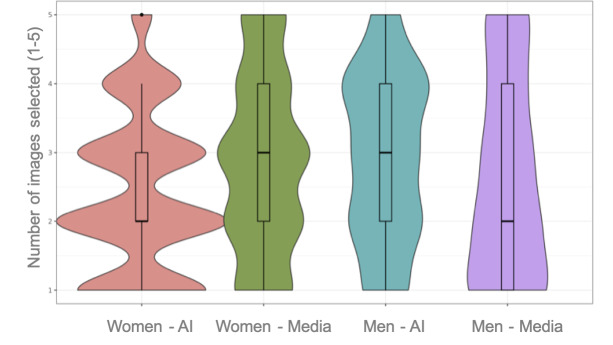
Violin plot for the question “Which images represent depression more realistically?” The Y axis represents the amount of images selected, from 1 to 5. The median for each column is marked with a thicker black line. We can observe how, out of the 5 possible options to select, women had a median of 2 artificial intelligence (AI) images (widest curve of the first column), as opposed to a median of 3 media images (widest curve of the second column). Differences among men were not statistically significant.

Responses to positively framed questions from people with depression were crossed with their demographic data. In terms of sex, no significant differences were found regarding which images were more appropriate (*χ*^2^_1_=1.785; *P*=.18; φ=.02), more inclusive (*χ*^2^_1_=0.289; *P*=.59; φ=0), and better represented the relationship between gender and depression (*χ*^2^_1_=3.430; *P*=.06; φ=.04). However, when they were asked about which images represented depression more realistically, women chose media images more frequently (*χ*^2^_1_=7.877; *P*=.005; φ=.06) Figure 2).

Regarding age groups, no significant differences were found in their preference for media or AI-generated images when asked about what images were more realistic (*χ*^2^_5_=6.439; *P*=.27; V=0.03) and appropriate (*χ*^2^_5_=7.4996; *P*=.19; V=0.04) to represent depression. However, when asked about which images better represented the relationship between gender and depression, 25- to 29-year-olds and 40- to 50-year-olds showed significant differences in selecting media images more frequently (*χ*^2^_1_=13.211; *P*<.001; φ=.11 and *χ*^2^_1_=28.9; *P*<.001; φ=.08, respectively). Likewise, when asked about which images were more inclusive, significant differences were found among 35- to 39-year-olds, who chose media images more frequently (*χ*^2^_1_=13.111; *P*=.002; φ=.17).

The same tests were conducted for young people. In terms of sex, statistically significant differences were observed when asked which were more appropriate to represent depression, with women choosing media images more frequently (*χ*^2^_1_=15.085; *P*<.001; φ=.09). There were also significant differences when asked about which images were more realistic; in this case, both men and women preferred media images, but women chose media images at a much higher rate (*χ*^2^_1_=152.4932; *P*<.001; φ=.13) than men did (*χ*^2^_1_=4.5533; *P*=.03; φ=.04).

#### Responses to Negatively Framed Questions

The questions that were framed negatively asked which images were considered more stereotyping, which images reproduced marginalization or social exclusion stigmas, and which images could affect people with depression more negatively.

A nonparametric Pearson chi-square test was used to examine differences between the test and control groups among people with depression. There were statistically significant differences between the 2 groups (*P*<.001): while the test group chose media images more frequently (1527/2712, 56.31% chose media; 1185/2712, 43.69% chose AI), the control group prioritized AI-generated images (1042/2733, 38.13% chose media; 1691/2733, 61.87% chose AI). Although these results follow the same pattern as those observed in positively framed questions, here the test group selected AI-generated images at a higher ratio.

**Figure 3.**
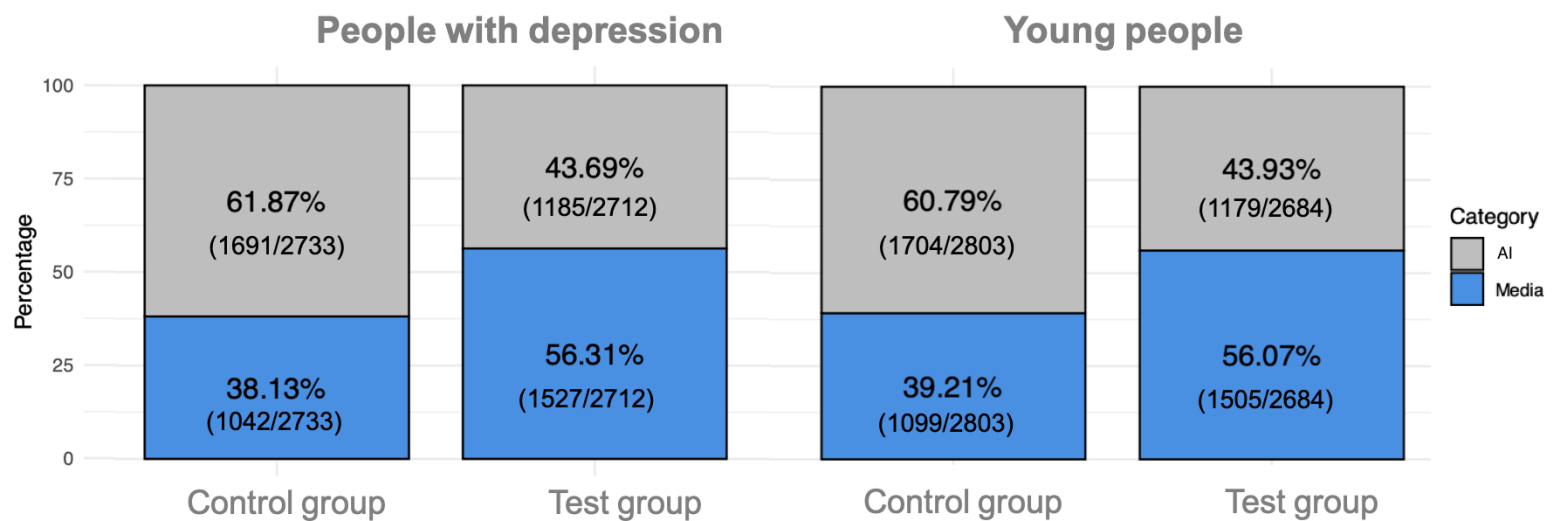
Distribution of answers for negatively framed questions regarding media or artificial intelligence (AI)–generated images, segregated by group among people with depression and young people.

A similar trend was observed among young people’s responses. There were statistically significant differences between the test and control groups (*P*<.001)—while the test group selected media images more frequently (1505/2684, 56.07% chose media; 1179/2684, 43.93% chose AI), the control group preferred AI-generated images (1099/2803, 39.21% chose media; 1704/2803, 60.79% chose AI; Figure 3).

The top 3 images selected for negatively framed questions among people with depression were all from the media among the test group, whereas the control group selected only AI-generated images in the top 3 positions. Among young people, the top 3 images for negatively framed questions followed a similar pattern: all the top 3 images among the test group were from the media, while 2 out of the top 3 images of the control group were AI-generated. Among people with depression, the test group selected in the first place a media image of a woman sitting down, with her elbows to her knees, holding her head with her hands, looking down. The control group selected an AI-generated image of a young man sitting in the street, holding his knees with his arms, facing down. Among young people, the test group selected in the first place a media image of a back-lit silhouetted woman taking a pill. This control group chose the same image as the control group of people with depression. Images are available as MultimediaAppendices 2  3.

Responses to negatively framed questions from people with depression were crossed with their demographic data. No significant differences were found on the basis of sex regarding which images were more stereotyped (*χ*^2^_1_=0.02806; *P*=.87; φ=0), reproduced more stigmas (*χ*^2^_1_=0.59149; *P*=.44; φ=0) or were more likely to have a negative impact on people with depression (*χ*^2^_1_=0.51907; *P*=.47; φ=0). Likewise, no significant differences were found on the basis of age for those same questions (*χ*^2^_5_=2.8295, *P*=.73, V=0; *χ*^2^_5_=10.114, *P*=.07, V=0.05; and *χ*^2^_5_=8.6723, *P*=.12, V=0.05; respectively).

**Figure 4.**
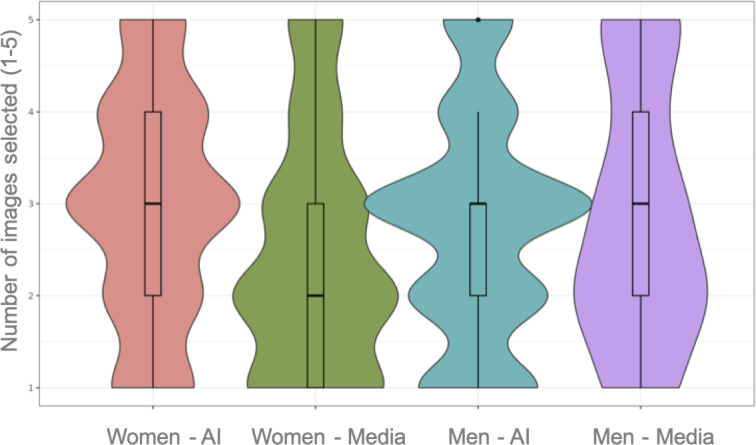
Violin plot for the question “Which images are more stereotyped?” The Y axis represents the amount of images selected, from 1 to 5. The median for each column is marked with a thicker black line. We can observe how, out of the 5 possible options to select, women had a median of 3 artificial intelligence (AI) images (widest curve of the first column), as opposed to a median of 2 media images (widest curve of the second column). Differences among men were not statistically significant.

The same analysis was conducted for young people’s responses. Significant differences were found between sexes (*χ*^2^_1_=9.6555; *P*=.002; φ=.07), with women choosing AI images more frequently when asked which images were more stereotyped (*χ*^2^_1_=18.2141; *P*<.001; φ=.04; Figure 4).

### How Do People With Depression Recommend Better Illustrating Depression, and How Do These Recommendations Compare to Those by Young People?

Respondents were asked to select their preferred sentences to help improve the way depression was depicted and were given the following five options: (a) show people with depression who are going about their life, spending time with family, friends, at work, having fun, at an everyday activity, and forth; (b) show more diversity of people in the images (different skin color, gender, age, functional diversity, types of bodies, etc); (c) show that help is available and that there are options for therapy, support, and accompaniment; (d) consider that depression has many degrees and that a single image cannot illustrate all it represents; and (e) consult with mental health specialists when choosing images.

**Figure 5.**
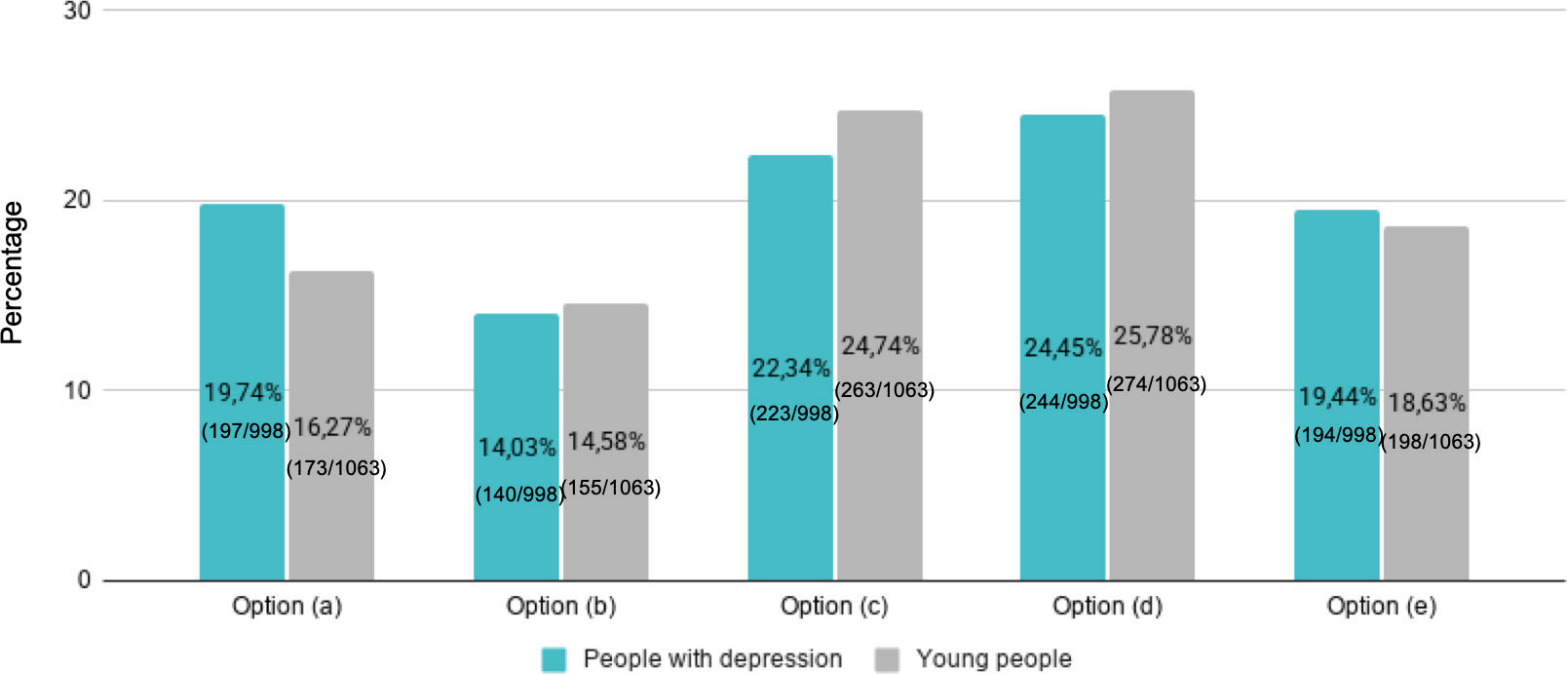
Double bar chart with respondents’ opinions (options a to e) regarding recommendations. Options: (a) show people with depression who are going about their life, spending time with family, friends, at work, having fun, at an everyday activity, and so forth; (b) show more diversity of people in the images (different skin color, gender, age, functional diversity, types of bodies, etc); (c) show that help is available and that there are options for therapy, support, and accompaniment; (d) consider that depression has many degrees and that a single image cannot illustrate all it represents; and (e) consult with mental health specialists when choosing images.

Both people with depression and young people selected option (4) in the first place, “consider that depression has many degrees and that a single image cannot illustrate all it represents” (people with depression: 244/998, 24.45%; young people: 274/1063, 25.78%). In second place, both groups also agreed with option (3), “show that help is available and that there are options for therapy, support, and accompaniment” (people with depression: 223/998, 22.34%; young people: 263/1063, 24.74%). In third place, people with depression chose option (1), “show people with depression who are going about their life, spending time with family, friends, at work, having fun, at an everyday activity, and so forth” (197/998, 19.74%), while young people chose option (5), “consult with mental health specialists when choosing images” (198/1063, 18.63%; Figure 5).

Among people with depression, there were no statistically significant differences between the test and control groups ((a) odds ratio [OR]=0.96, 95% CI 0.62-1.47; *P*=.84; (b) OR=0.89, 95% CI 0.58-1.38; *P*=.62; (c) OR=1.27, 95% CI 0.82-1.98; *P*=.29; (d) OR=1.01, 95% CI 0.64-1.58; *P*=.98; (e) OR=0.69, 95% CI 0.45-1.05; *P*=.08), nor when segregated by sex ((a) OR=1.22, 95% CI 0.76-1.99; *P*=.41; (b) OR=1.40, 95% CI 0.86-2.30; *P*=.17; (c) OR=1.29, 95% CI 0.79-2.08; *P*=.31; (d) OR=1.15, 95% CI 0.69-1.89; *P*=.59; (e) OR=0.74, 95% CI 0.46-1.19; *P*=.22). However, differences were observed among age groups for option (a), with 40- to 50-year-olds (OR=2.44, 95% CI 1.21-4.90; *P*=.004) and 25- to 29-year-olds (OR=3.73, 95% CI 1.18-11.78; *P*=.01) being more likely to select this option than people older than 51 years. Among young people, there were no statistically significant differences between the test and control groups ((a) OR=0.96, 95% CI 0.62-1.47; *P*=.84; (b) OR=0.89, 95% CI 0.58-1.38; *P*=.62; (c) OR=1.27, 95% CI 0.82-1.98; *P*=.29; (d) OR=1.01, 95% CI 0.64-1.58; *P*=.98; (e) OR=0.69, 95% CI 0.45-1.05; *P*=.08). However, there were significant differences between sexes, with female respondents choosing more frequently than male respondents options (b) (OR=2.14, 95% CI 1.19-3.98; *P*=.01) and (c) (OR=1.79, 95% CI 1.06-3.02; *P*=.03).

### How Do People With Depression, as Well as Young People, Receive Recommendations From Mental Health Associations Regarding How to Illustrate Depression?

Respondents were offered a scale from 0 to 5 to indicate the appropriateness of the images recommended by mental health associations (0=“images are very inadequate to represent depression” and 5=“images are very adequate to represent depression”).

**Figure 6.**
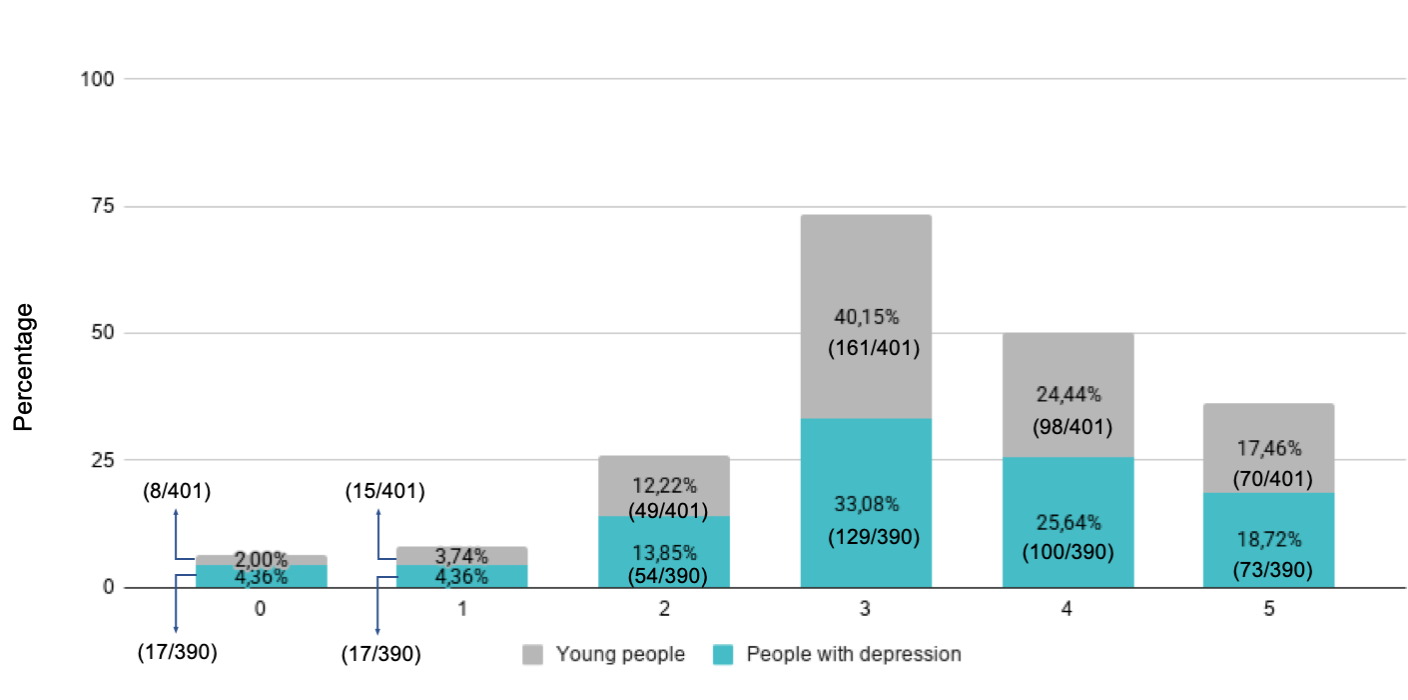
Stacked bar chart with responses (scale 0‐5) with respondents’ opinions regarding the appropriateness of images recommended by mental health associations.

Among people with depression, 44.36% (173/390) responded with ratings between 4 and 5. The most common single rating was 3 (129/390, 33.08%). The average (mean) was 3.27 (SD 1.28), and the median and mode were 3 (IQR 3-4). Young people responded in a very similar pattern: 41.90% (168/401) selected ratings between 4 and 5. The most common single rating was also 3 (161/401, 40.15%). The average (mean) was slightly higher than among people with depression, with 3.34 (SD 1.13), and the median and mode were also 3 (Figure 6).

There were no significant differences among people with depression depending on group (median test group 4, IQR 4-5 vs median control group 4, IQR 4-5; W=19134; *P*=.91), sex (median males 4, IQR 3-5 vs median females 4, IQR 4-5; W=14224; *P*=.19) or region (*χ*^2^_15_=18.936; *P*=.22). Likewise, for young people, no differences were found depending on group (median test group 4, IQR 4-5 vs median control group 4, IQR 4-5; W=20726.5; *P*=.57), sex (median males 4, IQR 4-5 vs median females 4, IQR 4-5; W=11397; *P*=.08) or region (*χ*^2^_16_=7.731; *P*=.96). These results were validated with the Mann-Whitney U test and the Kruskal-Wallis test. Although all age groups consider these recommendations positively, people with depression between the ages of 35 to 39 years do so the most.

## Discussion

### Principal Findings

This study analyzed media and AI-generated images representing depression and complemented that with an exploration of the perceptions of people with depression and young people regarding those images. This study also analyzed how recommendations from mental health associations regarding how depression should be illustrated are perceived by people with depression and young people. This research offers valuable insights into the quality and appropriateness of media and AI-generated images, indicating overt preferences among respondents. The integration of a quasi-experimental survey, including test and control groups, ensures the robustness of the results.

The first research question we set ourselves to address was “How is the representation of depression in media and AI-generated images perceived?” The results indicate that the representation of depression is perceived more negatively in images generated by AI than in images from the media. That is, AI-generated images are perceived as more stereotyping, that they reproduce stigmas of marginalization or social exclusion, and that they affect people with depression more negatively. In contrast, compared with AI-generated images, media images are perceived to be more appropriate, realistic, inclusive, and to better represent the relationship between gender and depression.

However, it must be noted that both media and AI-generated images reproduce stereotypes and stigma about depression. Most media and AI images include characteristics that associations suggest avoiding, such as depicting people alone; without social interactions; staring into space; lying on the floor; covering their faces; or in long, dark hallways [45].

The second research question was “How do the opinions of people with depression differ from the opinions of young people regarding the depiction of depression in media and AI-generated images?” In short, their answers follow a similar pattern in most questions. Let us take a deeper look into each of the groups.

According to the survey results, people with depression tend to favor media images over AI-generated images. This trend is observed particularly strongly in questions that are framed positively (in this case, asking which images are considered more appropriate, realistic, inclusive, and better represent the relationship between gender and depression). People with depression from the test group (ie, those who know which images are from the media and which are AI-generated) clearly prioritize media images over AI-generated ones in positively framed questions. In other words, people from the control group (ie, those who do not know which images are from the media and which are AI-generated) select AI-generated images at a higher ratio than those in the test group. Compared with young people’s responses, the same trend is observed: young people from the test group also prioritize media images, or avoid AI-generated images, in positively framed questions, while the difference is less pronounced in the control group. This supports the idea that when people know which images are AI-generated, they tend to reject them, indicating a negative preconception about AI. This finding coincides with previous research observing a prejudice against AI-generated products [41,42]. However, another interpretation could be that test group participants could identify the more stereotypical images because they had been told in advance which ones were AI-generated.

There are also negatively framed questions, asking which images are considered more stereotyping, reproduce marginalization or social exclusion stigmas, or can affect people with depression more negatively. For these types of questions, people with depression from the test group choose media images more frequently than AI-generated ones, while people from the control group prioritize AI-generated images over media ones. This pattern is the same among young people, who also tend to select a higher ratio of AI-generated images when they cannot distinguish them from the media ones. Interestingly, the differences between the test and control groups do not seem to be influenced by demographic variables, such as sex, age, or region.

It is relevant to note how the framing of a question affects its responses, coinciding with previous literature indicating that audiences’ reactions depend on the framing of images [17]. In this study, the results obtained from positively and negatively framed questions followed the same pattern: the test groups always prioritized media images, while the control group favored AI-generated images more frequently. However, in negatively framed questions, control groups select AI-generated images at a higher ratio than test groups do. This could indicate that when people know which images are generated by AI, they tend to associate them more strongly with negative concepts, such as stereotypes or stigma. This interpretation aligns with previous research, as mentioned before, observing a prejudice against AI-generated products [41,42].

The third research question of the study was as follows: “How do people with depression recommend better illustrating depression, and how do these recommendations compare to those by young people?” This question is particularly relevant if we consider that nowadays, AI-generated images do not depict mental health conditions accurately [30,31]; thus, the need for recommendations becomes clear. The survey results indicate that people with depression and young people share similar opinions regarding the proposed recommendations, as they both select the same options in first and second place (“consider that depression has many degrees and that a single image cannot illustrate all it represents,” followed by “show that help is available and that there are options for therapy, support, and accompaniment”). For these recommendations, no statistically significant differences are observed among people with depression, either by test and control groups or when segregated by demographic variables.

Among young people, no statistically significant differences are observed between test and control groups; however, when segregated by sex, women choose the second recommendation (“show that help is available and that there are options for therapy, support, and accompaniment”) more frequently than men do. This result is supported by the literature showing that men are less inclined than women to seek help for mental health issues [48,49].

The fourth and final research question to address was as follows: “How do people with depression, as well as young people, receive recommendations from mental health associations regarding how to illustrate depression?” Both respondent groups perceive these recommendations positively: from a scale of 0‐5 asking about the appropriateness of the images recommended, the average among people with depression is 3.27 (SD 1.28), which is slightly lower than the 3.34 (SD 1.13) among young people. These results indicate that both people with depression and young people approve of the recommendations provided by mental health associations. This positive result can be interpreted as validation of the recommendations.

The observed gender- and age-related differences in attitudes toward technology use can be interpreted through established theoretical frameworks, such as the TAM. The TAM proposes that perceived usefulness and perceived ease of use shape behavioral intentions, and these perceptions may vary across demographic groups due to differences in prior technological experience [39,40]. This model provides a theoretical basis for understanding why demographic characteristics can lead to systematic differences in technology acceptance, as observed in this study.

In conclusion, there is currently a clear trend toward the widespread use of AI in journalism [50], as well as in science communication [51]. It is not difficult to imagine a future in which AI-generated images will start to appear in the media, in the same way as stock images do today. In this scenario, it is of utmost importance to consider social perceptions and opinions, particularly those of the groups most affected by the use of the new technology. Moreover, and particularly because negative media representations of mental health lead to mental health stigma [14-16], the findings of our study underscore the need for closer collaboration among journalists, AI developers, mental health experts, and patient associations, as well as a shift toward user-participatory AI design. With this study, we aimed to contribute to these considerations by highlighting social perceptions regarding the use of AI tools in the media and providing enough data to guide a more responsible way forward for the benefit of all.

### Limitations

Despite the insights offered by this study, some limitations warrant consideration. First, we base our results on a selection of 30 images. Although it is clear that a limit to the number of images is needed to conduct the study, this same limit means that opinions and perceptions are inextricably linked to the images selected. It is possible that a different pool of images could produce dissimilar outcomes.

Second, the authors note that both AI technology and the ethical protocols that guide it evolve rapidly [52], as do people’s sentiments about it [53]. This could mean that, in a few years, AI-generated images could reproduce fewer stereotypes, or that people’s perceptions of AI-generated images have shifted toward a more positive outlook. With this in mind, this study would benefit from being repeated over time to monitor whether its results are sustained over time.

Third, the qualitative sample of the discussion groups is not gender-balanced (13 women, 2 men). Since the qualitative phase informed the quantitative one, the authors acknowledge that this gender imbalance may have biased the development of the survey questions.

Fourth, the authors note that the predominantly Spanish cultural context of the study may limit the transferability of results to other cultural environments, where different social norms and practices may be at play.

Fifth, there is a potential confound with study populations. The group “Young people” did not include individuals with depression; however, the group “People with depression” was not restricted by age and therefore also included young people. This constitutes a potential confound of the study populations.

Finally, a methodological limitation of the study is the use of a simplified prompt (“depression”) to generate the AI images. This single-word prompt could have increased the probability that generative models defaulted to widely learned, stereotypical visual representations of depression present in their training data. Therefore, the observed tendency for AI-generated images to appear “more stereotypical” could actually be a reflection of the prompting strategy rather than an inherent shortcoming of the generative systems themselves.

### Conclusions

Academic literature had reported the impact of AI on mental health communication, but the effectiveness, limitations, and ethical issues of using AI in the communication of mental health, particularly depression, were yet to be studied. Combining qualitative and quantitative approaches, our study offers novel insights into how people with depression and young people perceive the use of AI to generate images of depression. The results show that although both media and AI-generated images are considered to reproduce stereotypes about depression, respondents tend to reject AI-generated images, indicating a certain bias against AI-produced pictures. These results hold substantial implications in the current drift toward the widespread use of AI in mental health communication, highlighting the need to build bridges among science journalists, AI developers, mental health experts, and patient associations.

## Supplementary material

10.2196/81230 Multimedia Appendix 1 Distribution of the discussion groups organized, specifying their date, time, profile of participants, mode of attendance, and number and gender of participants.

10.2196/81230 Multimedia Appendix 2 Media images.

10.2196/81230 Multimedia Appendix 3 Artificial intelligence (AI)–generated images.

10.2196/81230 Multimedia Appendix 4 Surveys.

## Abbreviations

AI: artificial intelligence
OR: odds ratio
RQ: research question
TAM: technology acceptance model
