# Supplementary material for: Comparing Images of Depression in Mass Media and AI-Generated Pictures: Mixed Methods Study
Source: JMIR Hum Factors. 2026 Apr 14;13:e81230. doi: 10.2196/81230 (PMC13094379; doi:10.2196/81230)
Supplement: Multimedia Appendix 1 [file humanfactors-v13-e81230-s001.docx]

**Supplementary material 1**

| ID | Date | Time | Profile | Attendance | Participants | Gender |
| --- | --- | --- | --- | --- | --- | --- |
| 1 | 11/01 | 13:00-15:00 | People with depression | Online | 5 | 4 women  1 man |
| 2 | 15/01 | 16:00-18:00 | Young people | Hybrid | 7 | 6 women  1 man |
| 3 | 18/01 | 13:30-15:00 | Young people | Face to face | 3 | 3 women  0 men |

Distribution of the discussion groups organized, specifying their date, time, profile of participants, mode of attendance, number and gender of participants.
