# Supplementary material for: Comparing Images of Depression in Mass Media and AI-Generated Pictures: Mixed Methods Study
Source: JMIR Hum Factors. 2026 Apr 14;13:e81230. doi: 10.2196/81230 (PMC13094379; doi:10.2196/81230)
Supplement: Multimedia Appendix 2 [file humanfactors-v13-e81230-s002.docx]

**Supplementary material 1**

Media images

| **Date** | **Newspaper** |
| --- | --- |
| ***Included in the survey*** | |
| [12/03/2023](https://www.eldiario.es/era/principales-sintomas-de-la-depresion-silenciosa_1_10007492.html) | *Eldiario.es* |
| [29/11/2023](https://www.larazon.es/gente/celebrities/shannen-doherty-empeora-llegar-cancer-sus-huesos-quiero-morir_20231129656797b7533bd200010e2f51.html) | *La Razón* |
| [03/07/2023](https://www.eldiario.es/sociedad/mitad-estudiantado-universitario-sintomas-depresion-ansiedad_1_10359148.html) | *Eldiario.es* |
| [27/02/2023](https://elpais.com/salud-y-bienestar/nosotras-respondemos/2023-02-27/cuales-son-los-sintomas-de-la-depresion-en-adolescentes.html?rel=buscador_noticias) | *El País* |
| [16/11/2023](https://www.larazon.es/tusalud/estudio-revela-que-determinado-tipo-alimentos-esta-asociado-depresion_2023111665565b3a32499c000145ff0f.html) | *La Razón* |
| [13/12/2023](https://www.larazon.es/gente/celebrities/dos-anos-veronica-forque-muerte-que-ayudo-cambiar-discurso-salud-mental_202312136579878f0ec7c80001e59dbf.html) | *La Razón* |
| [19/02/2023](https://elpais.com/opinion/2023-02-19/cual-es-la-otra-pandemia-que-nos-esta-matando.html?rel=buscador_noticias) | *El País* |
| [02/12/2023](https://www.larazon.es/salud/rumiar-problemas-causa-30-depresiones_2023120265699d2a0ec7c80001b19d4f.html) | *La Razón* |
| [30/11/2023](https://www.larazon.es/gente/casa-real/mary-dinamarca-habla-primera-vez-depresion_202311306568423991707700010e8712.html) | *La Razón* |
| [13/01/2023](https://www.eldiario.es/canariasahora/lapalmaahora/opinion/dia-mundial-lucha-depresion_129_9863369.html) | *Eldiario.es* |
| [15/05/2023](https://elpais.com/gente/2023-05-15/simon-pegg-durante-el-rodaje-de-mision-imposible-iii-mantuve-mi-alcoholismo-en-secreto.html?rel=buscador_noticias) | *El País* |
| [02/05/2023](https://www.eldiario.es/edcreativo/diario-salud/son-senales-advertencia-depresion-posparto_1_10150466.html) | *Eldiario.es* |
| [16/01/2023](https://elpais.com/salud-y-bienestar/2023-01-16/la-ciencia-busca-nuevos-metodos-para-combatir-la-depresion-deteccion-precoz-de-los-riesgos-y-tratamientos-individualizados.html?rel=buscador_noticias) | *El País* |
| [25/12/2022](https://elpais.com/icon/2022-12-25/fermin-muguruzareferente-de-un-tipo-de-masculinidad-yo-seguro-del-movimiento-antifa-puede.html?rel=buscador_noticias) | *El País* |
| [04/10/2023](https://elpais.com/economia/2023-10-04/la-brecha-de-genero-afecta-a-la-salud-emocional.html?rel=buscador_noticias) | *El País* |
| ***Not included in the survey, but included in discussion groups*** | |
| [04/12/2023](https://www.larazon.es/sociedad/que-navidad-pone-triste_20231204656d8103d6703700018e8c1f.html) | *La Razón* |
| [01/12/2023](https://www.larazon.es/economia/90-trabajadores-generacion-siente-ansiedad-trabajo_20231201656a007562c50d0001aac37d.html) | *La Razón* |
| [30/11/2023](https://elpais.com/gente/2023-11-30/mary-de-dinamarca-confiesa-por-primera-vez-que-sufrio-depresion.html?rel=buscador_noticias) | *El País* |
| [14/11/2023](https://www.larazon.es/sociedad/desafio-reducir-suicidio-jovenes_202311146553c6ffb276150001b1fbcd.html) | *La Razón* |
| [24/10/2023](https://elpais.com/mamas-papas/actualidad/2023-10-24/es-suficiente-una-pastilla-para-tratar-la-depresion-posparto.html?rel=buscador_noticias) | *El País* |
| [24/10/2023](https://www.larazon.es/television/daniela-santiago-protagonista-veneno-hace-publica-depresion-que-sufre-tonta-buena-deja-serlo-cuando-aura-sus-heridas_202310246537c20ea1cbe00001811589.html) | *La Razón* |
| [11/10/2023](https://www.larazon.es/gente/famosos/lucia-rivera-sus-problemas-salud-mental-hay-dias-que-puedo-levantar-cama_202310116526ce13e0d76200012617b8.html) | *La Razón* |
| [09/07/2023](https://elpais.com/icon/cultura/2023-07-09/linda-hamilton-la-luchadora-que-saco-fuerzas-de-sarah-connor-para-enfrentarse-a-sus-adicciones-y-al-trastorno-bipolar.html?rel=buscador_noticias) | *El País* |
| [13/05/2023](https://www.eldiario.es/sociedad/traumas-psicologicos-infancia-multiplican-cuatro-riesgo-depresion_1_10220000.html) | *Eldiario.es* |
| [23/02/2023](https://elpais.com/gente/2023-02-23/kylie-jenner-detalla-como-vivio-su-depresion-postparto-piensas-que-nunca-seras-la-misma.html?rel=buscador_noticias) | *El País* |
| [16/02/2023](https://www.eldiario.es/politica/senador-estadounidense-john-fetterman-hospitalizado-depresion_1_9960892.html) | *Eldiario.es* |
| [13/02/2023](https://www.eldiario.es/sociedad/tecnicas-aprendizaje-automatico-permiten-identificar-depresion_1_9948398.html) | *Eldiario.es* |
| [13/01/2023](https://www.eldiario.es/sociedad/no-porte-mal-le-nino-depresion_1_9863031.html) | *Eldiario.es* |
| [13/01/2022](https://www.eldiario.es/canariasahora/lapalmaahora/sociedad/dia-mundial-lucha-depresion_1_8651690.html) | *Eldiario.es* |
| [04/01/2019](https://www.eldiario.es/canariasahora/sociedad/ansiedad-depresion-sanidad-suicidio_1_1785782.html) | *Eldiario.es* |
