# Supplementary material for: Comparing Images of Depression in Mass Media and AI-Generated Pictures: Mixed Methods Study
Source: JMIR Hum Factors. 2026 Apr 14;13:e81230. doi: 10.2196/81230 (PMC13094379; doi:10.2196/81230)
Supplement: Multimedia Appendix 3 [file humanfactors-v13-e81230-s003.docx]

**Supplementary material 3**

AI-generated images

| **IA platform** | **Image** |
| --- | --- |
| ***Included in the survey*** | |
| Deep Dream Generator | 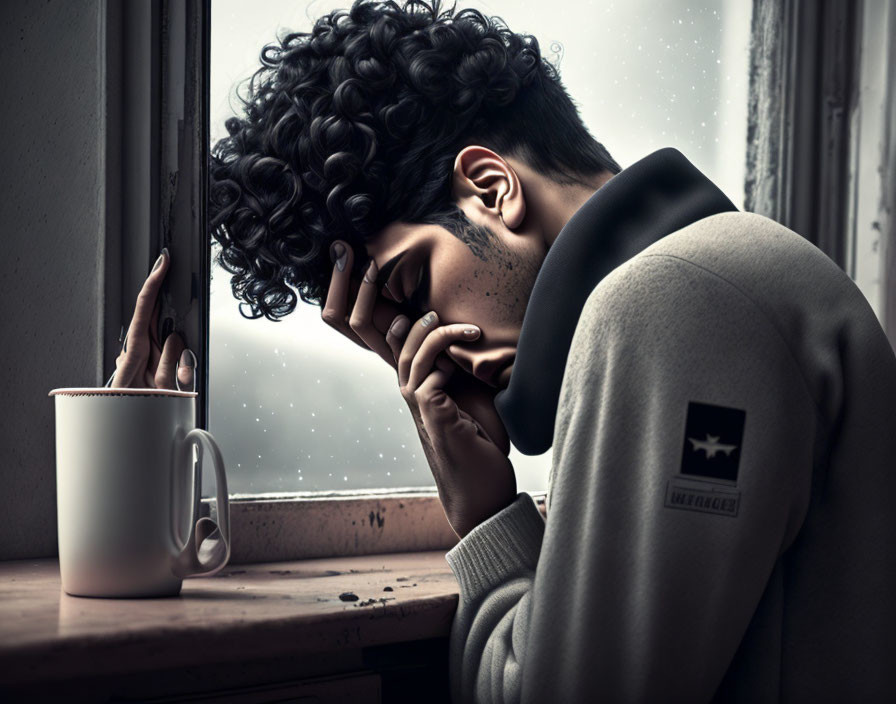 |
| Dream Studio | 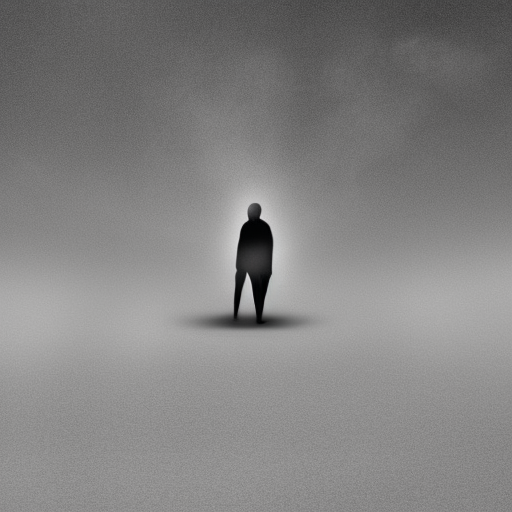 |
| Deep Dream Generator | 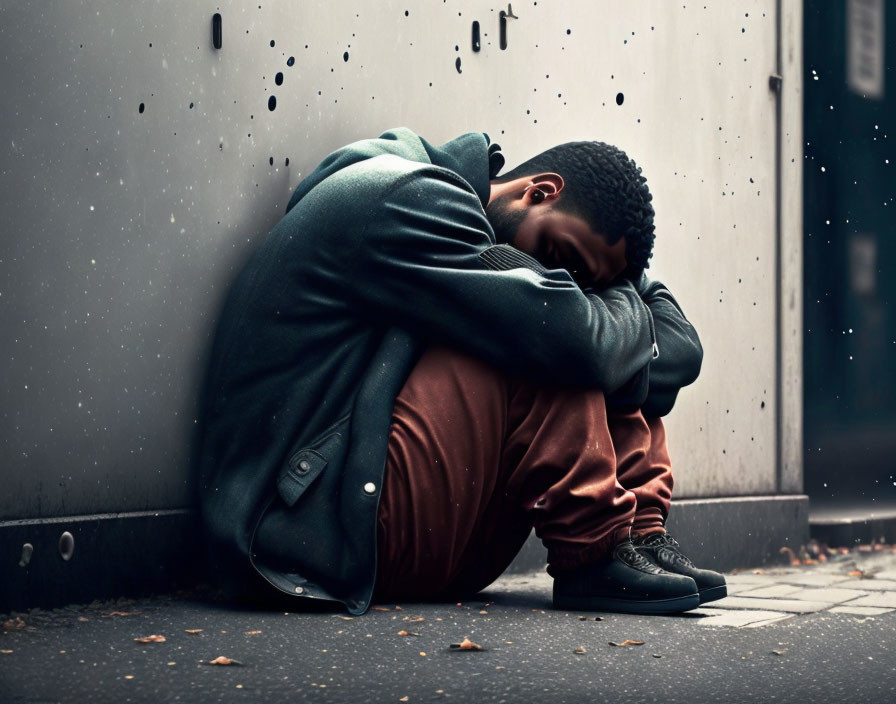 |
| DeepAI | 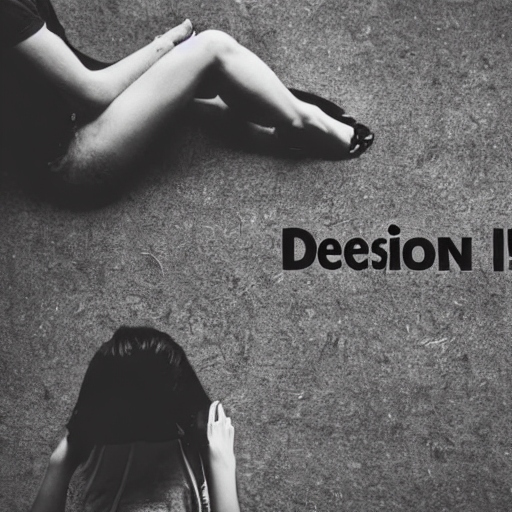 |
| DeepAI | 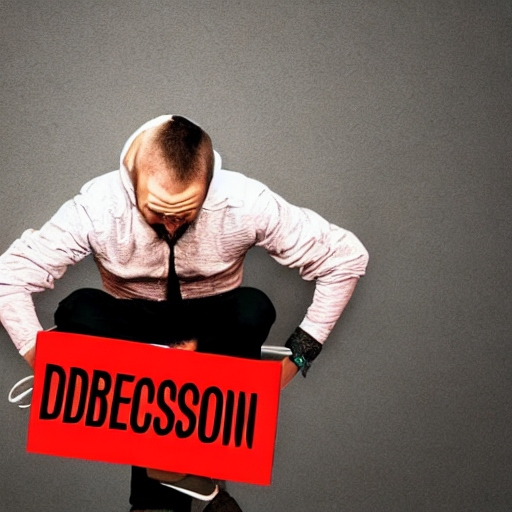 |
| OpenAI | 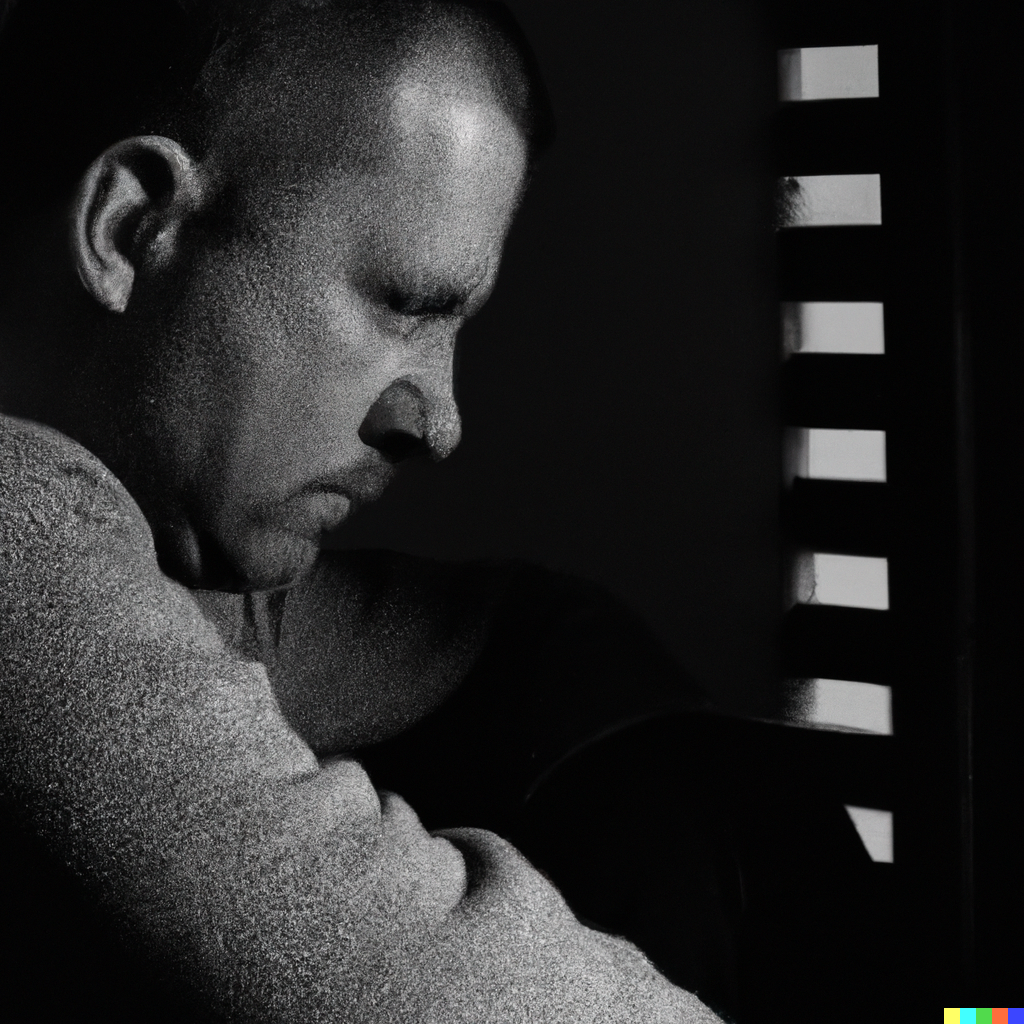 |
| OpenAI | 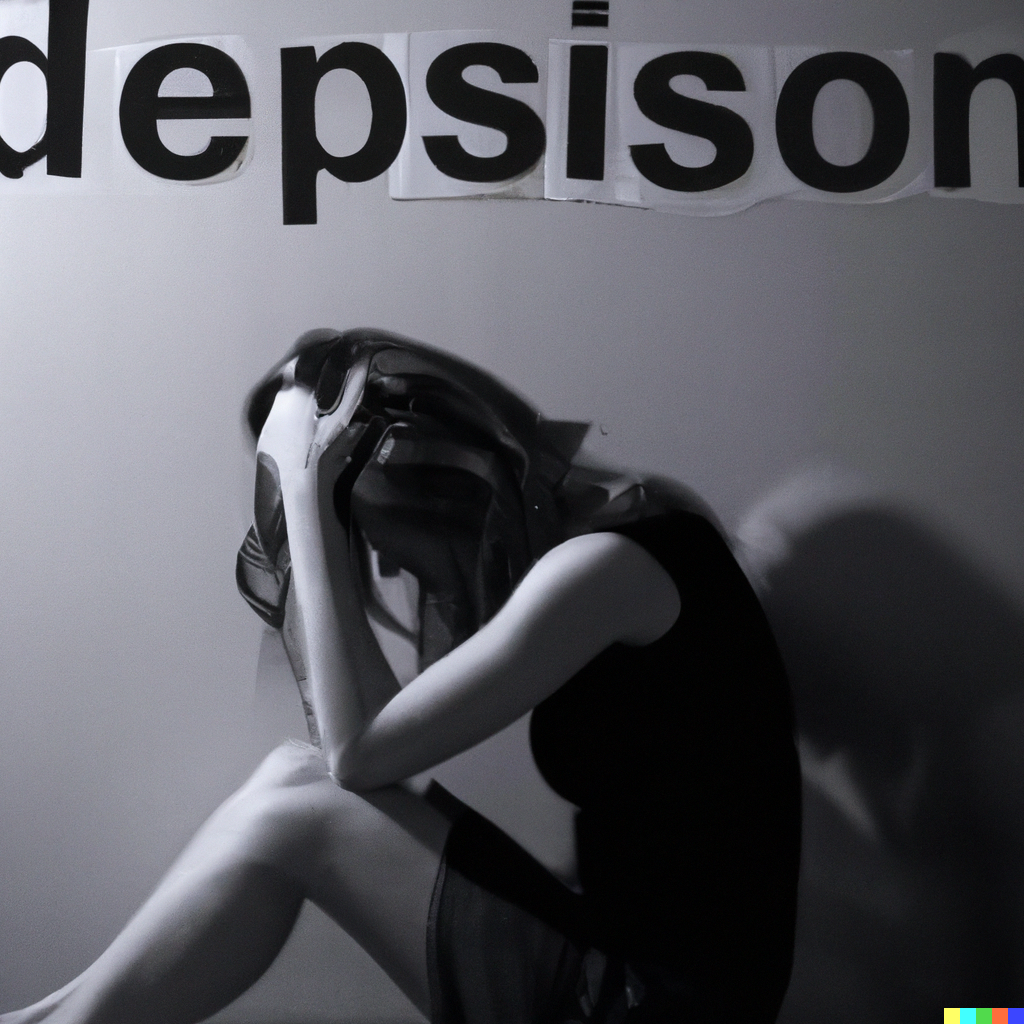 |
| DeepAI | 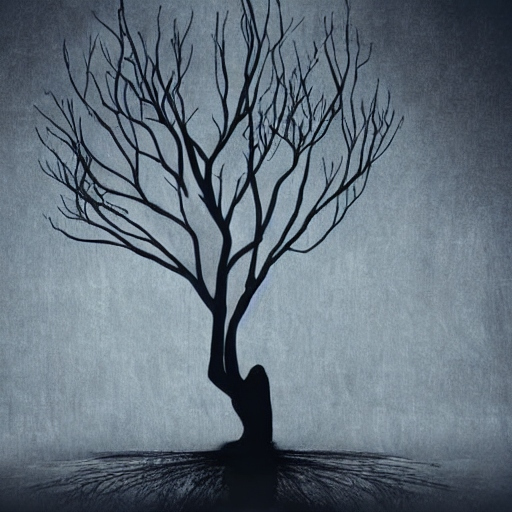 |
| Dream Studio | 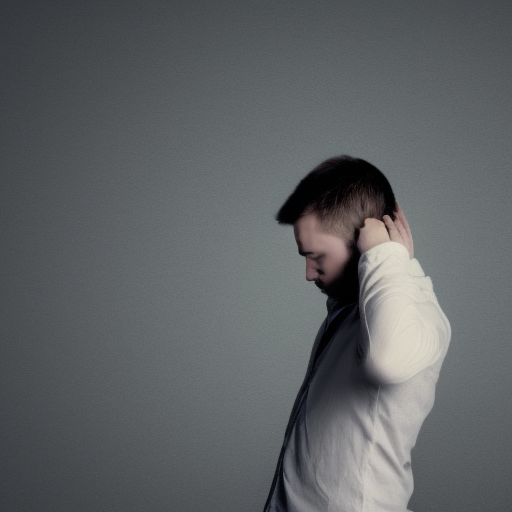 |
| OpenAI | 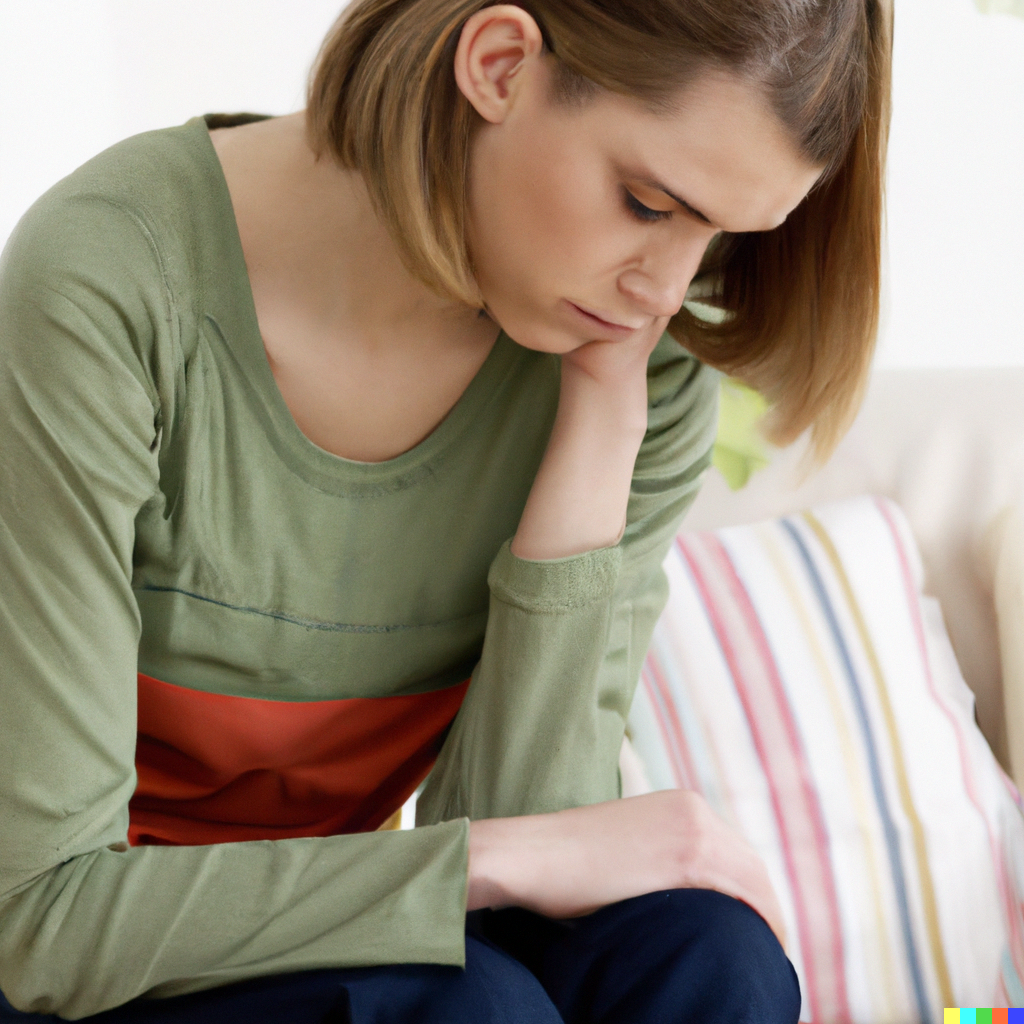 |
| DeepAI | 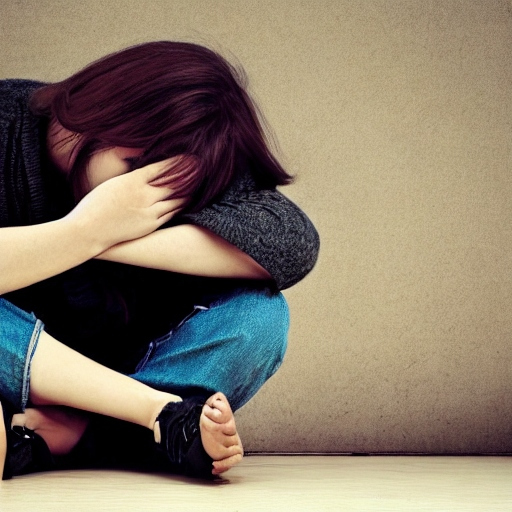 |
| Deep Dream Generator | 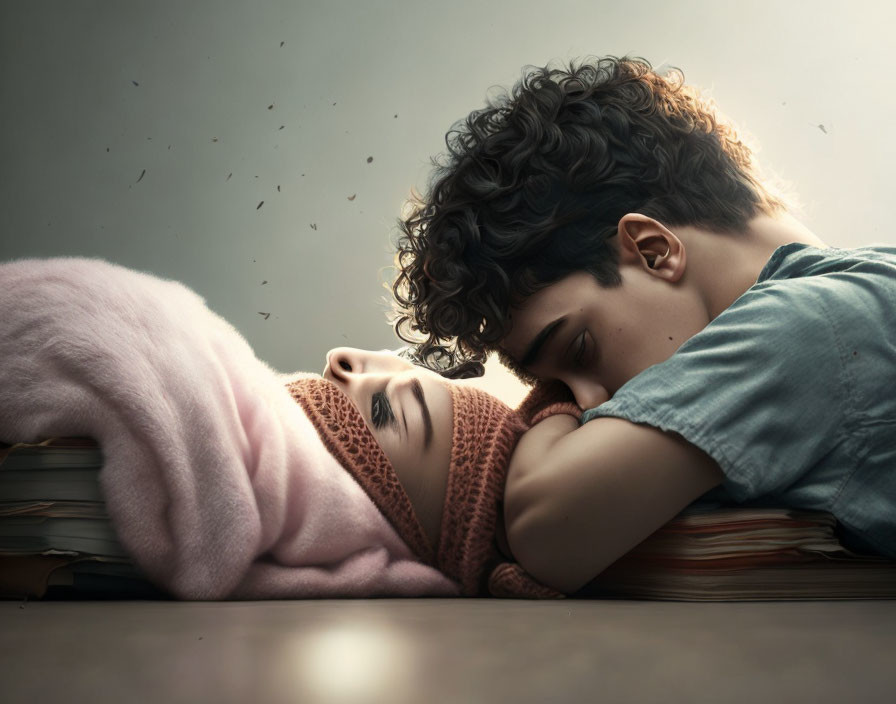 |
| Dream Studio | 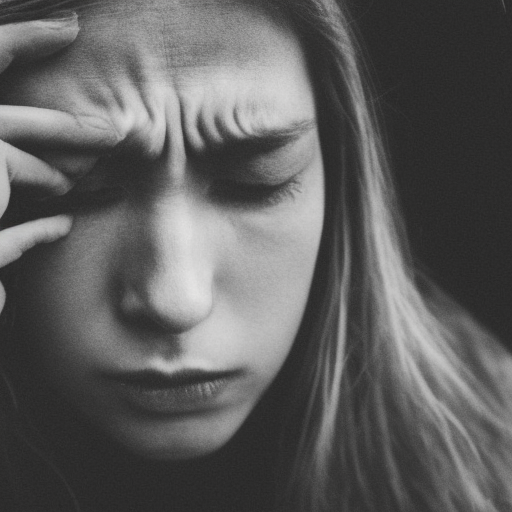 |
| OpenAI | 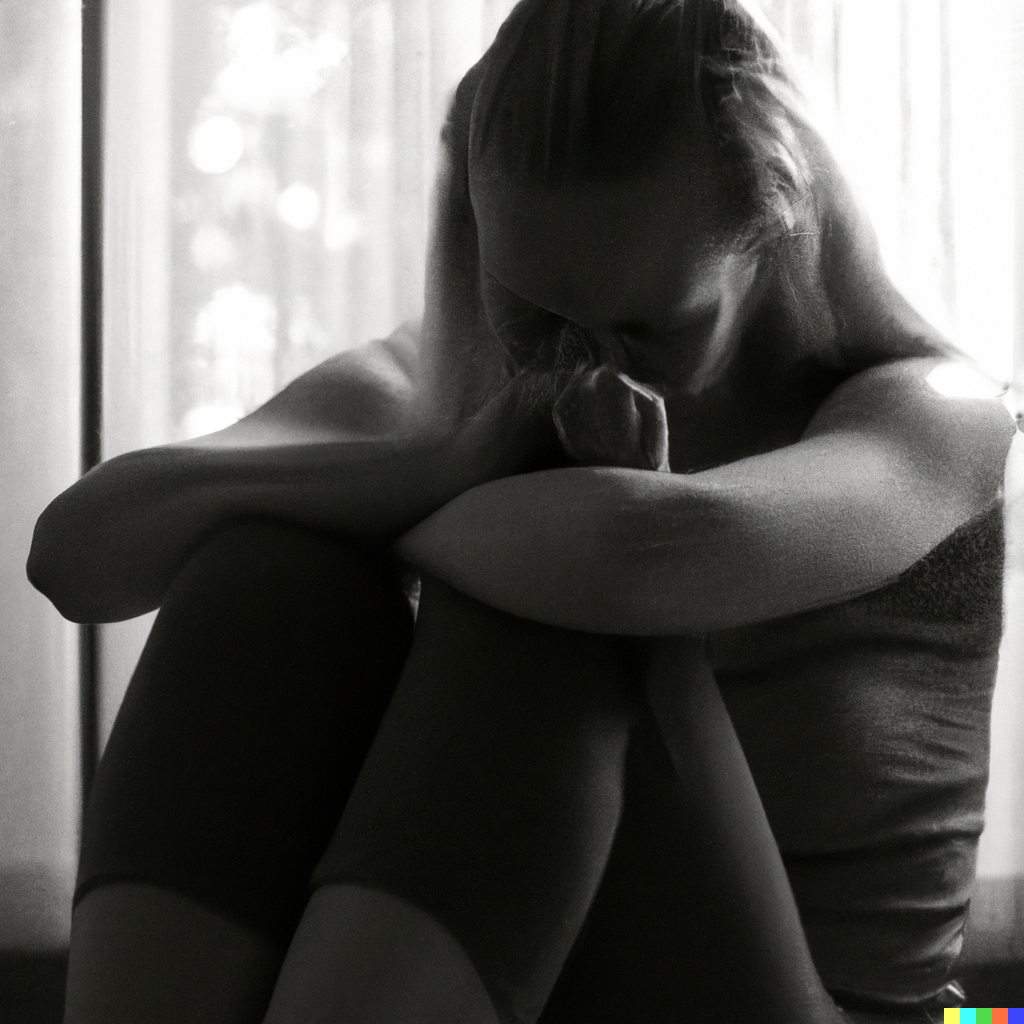 |
| Deep Dream Generator | 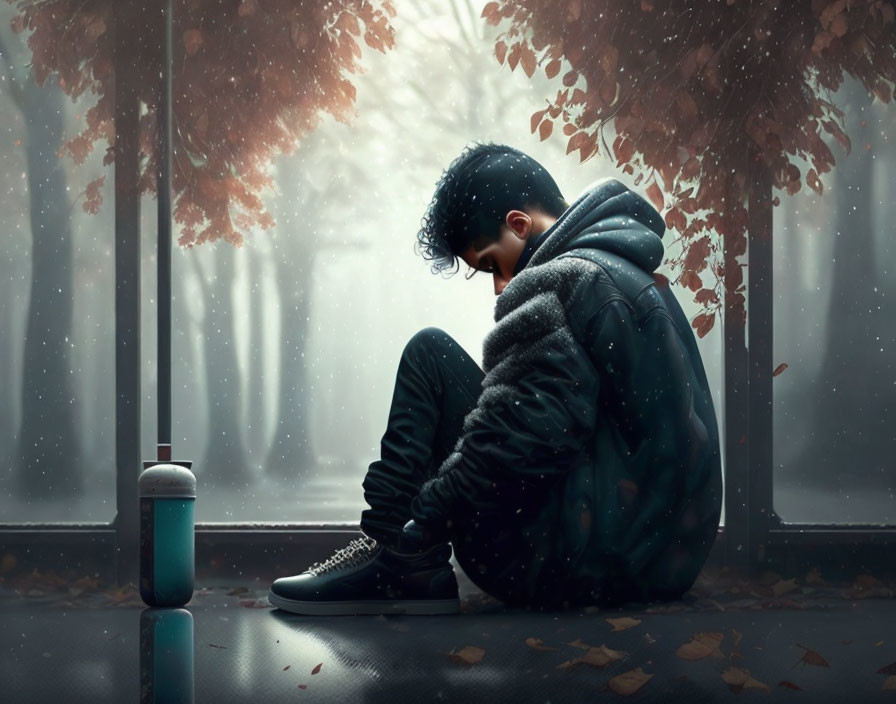 |
| ***Not included in the survey, but included in discussion groups*** | |
| Deep Dream Generator | 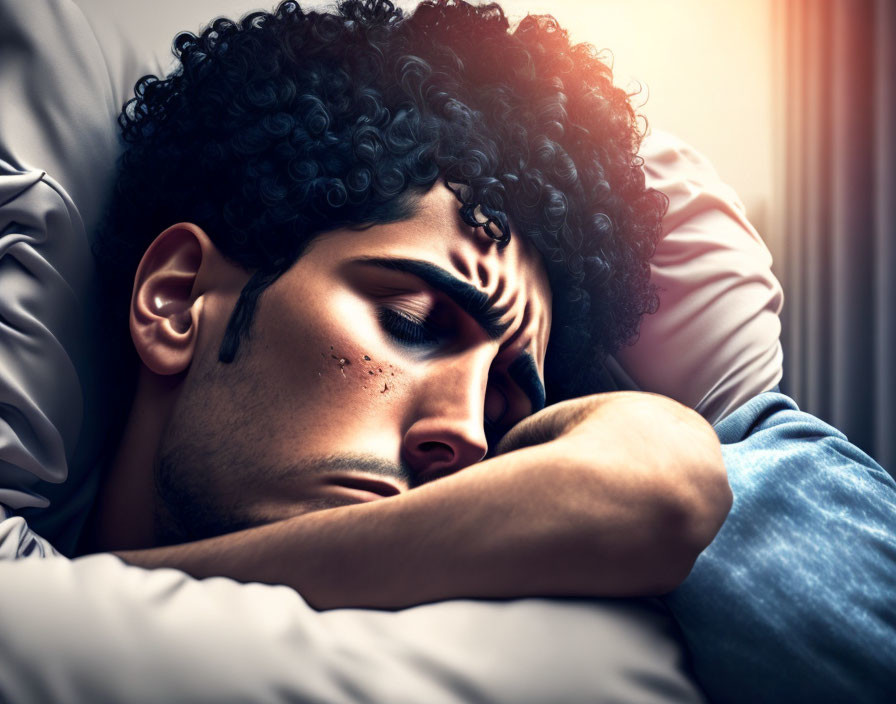 |
| Deep Dream Generator | 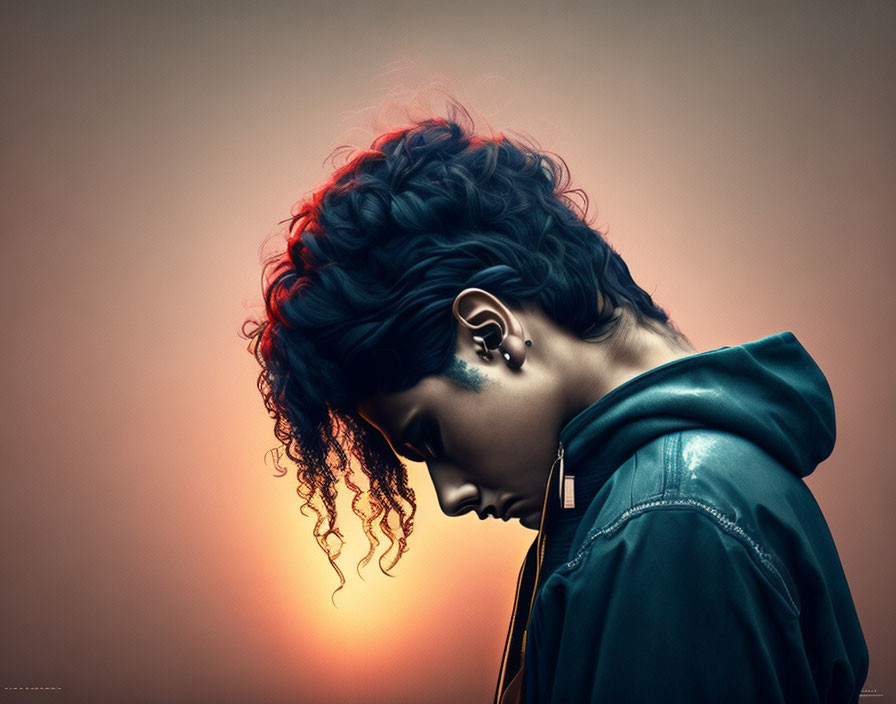 |
| Deep Dream Generator | 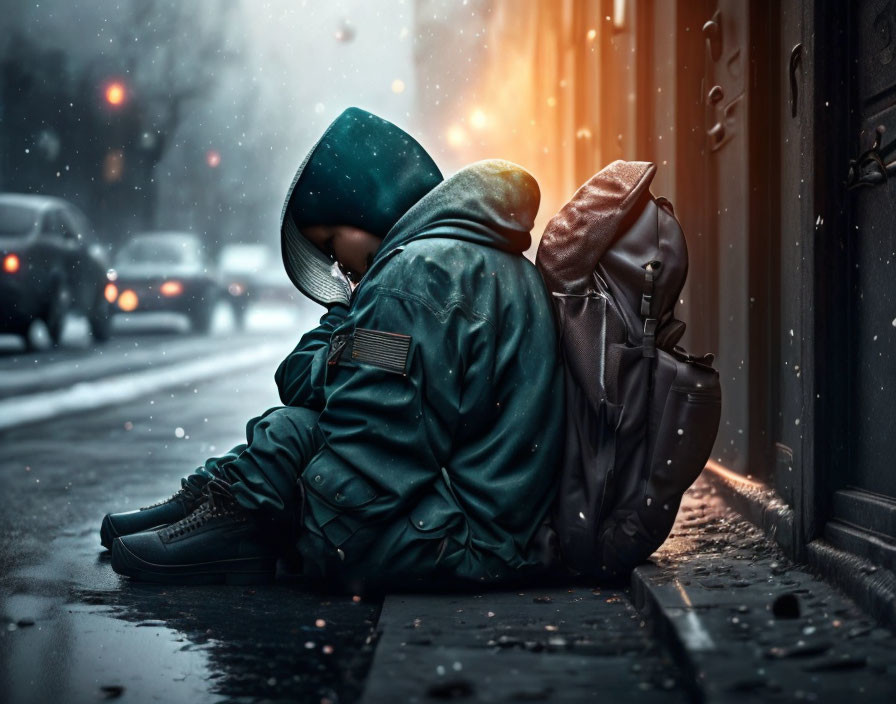 |
| Deep Dream Generator | 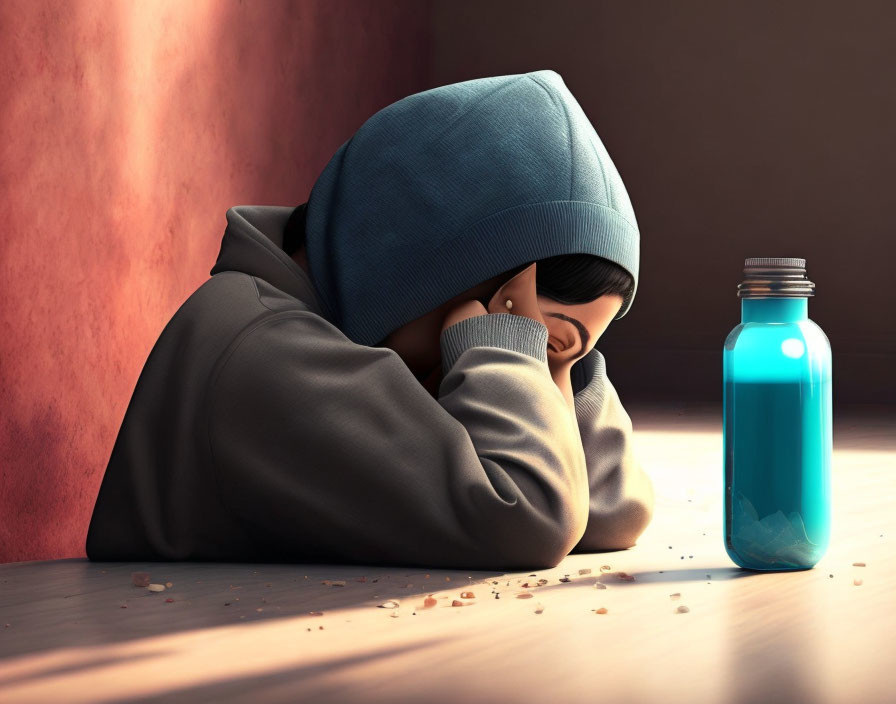 |
| Deep Dream Generator | 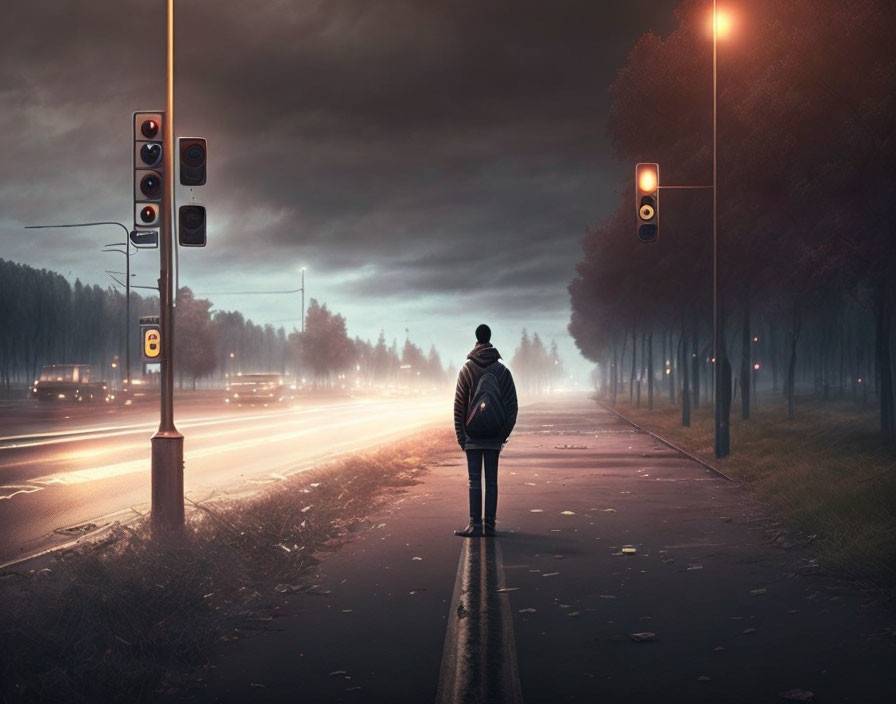 |
| DeepAI | 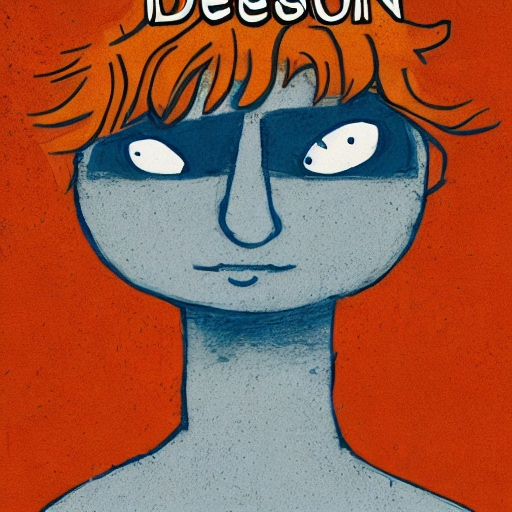 |
| DeepAI | 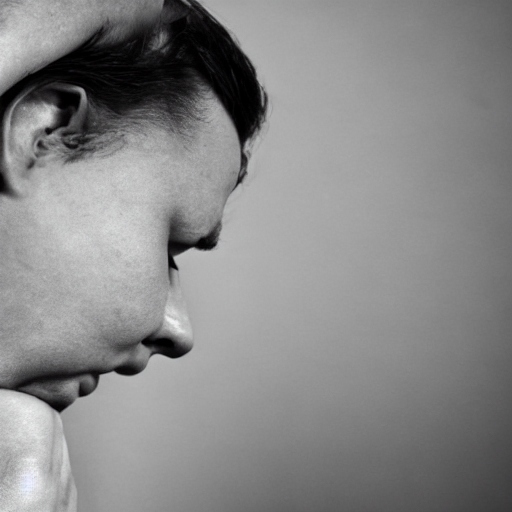 |
| DeepAI | 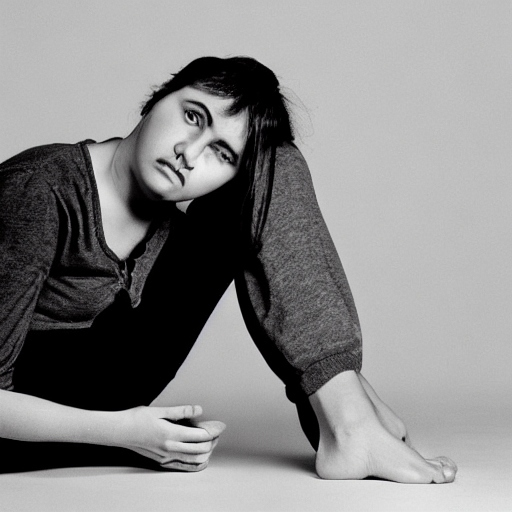 |
| OpenAI | 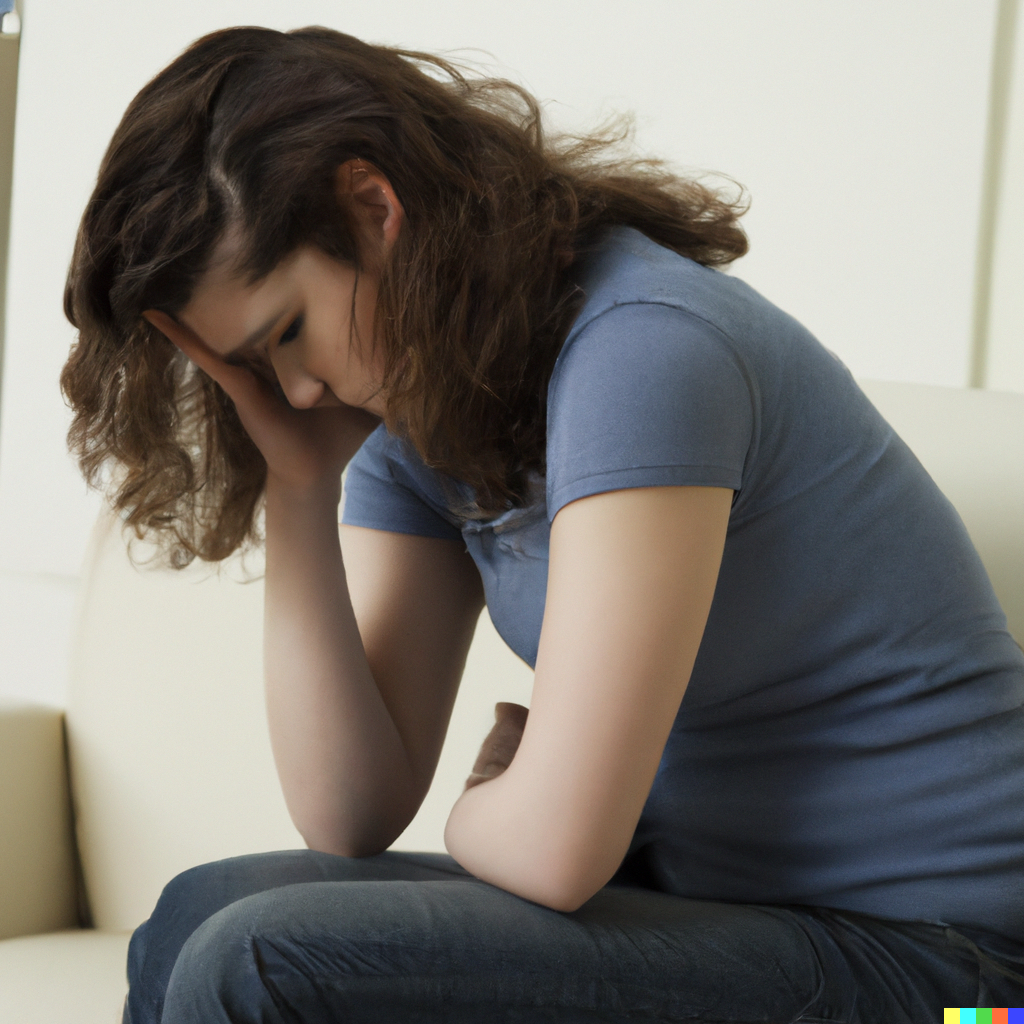 |
| OpenAI | 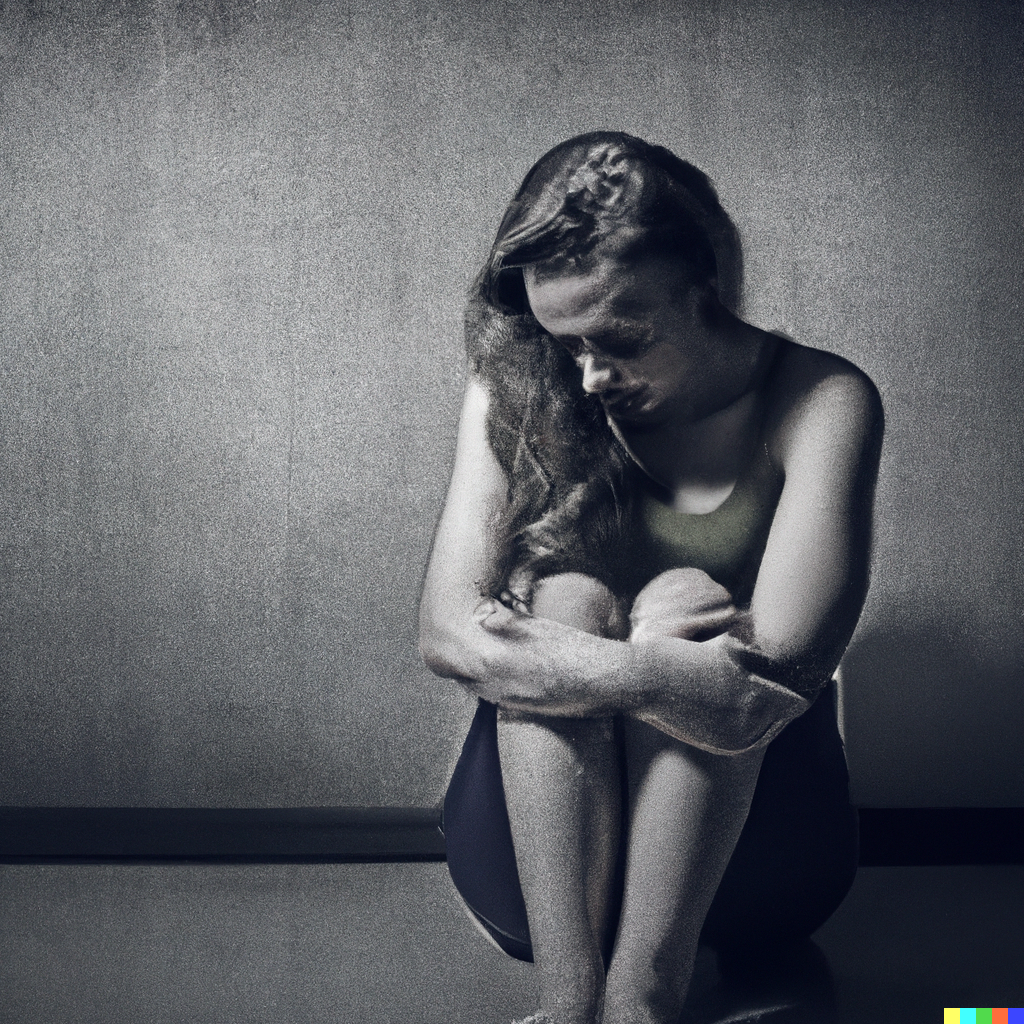 |
| OpenAI | 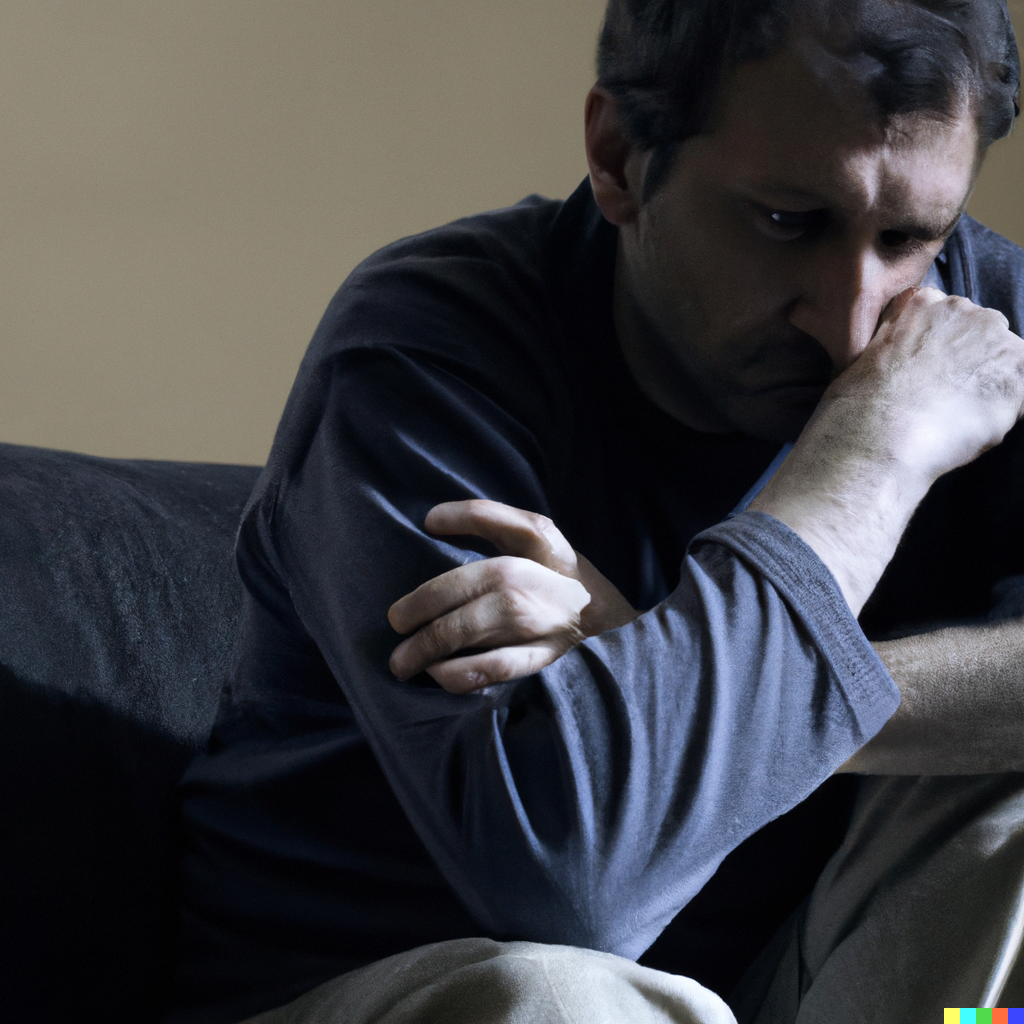 |
| OpenAI | 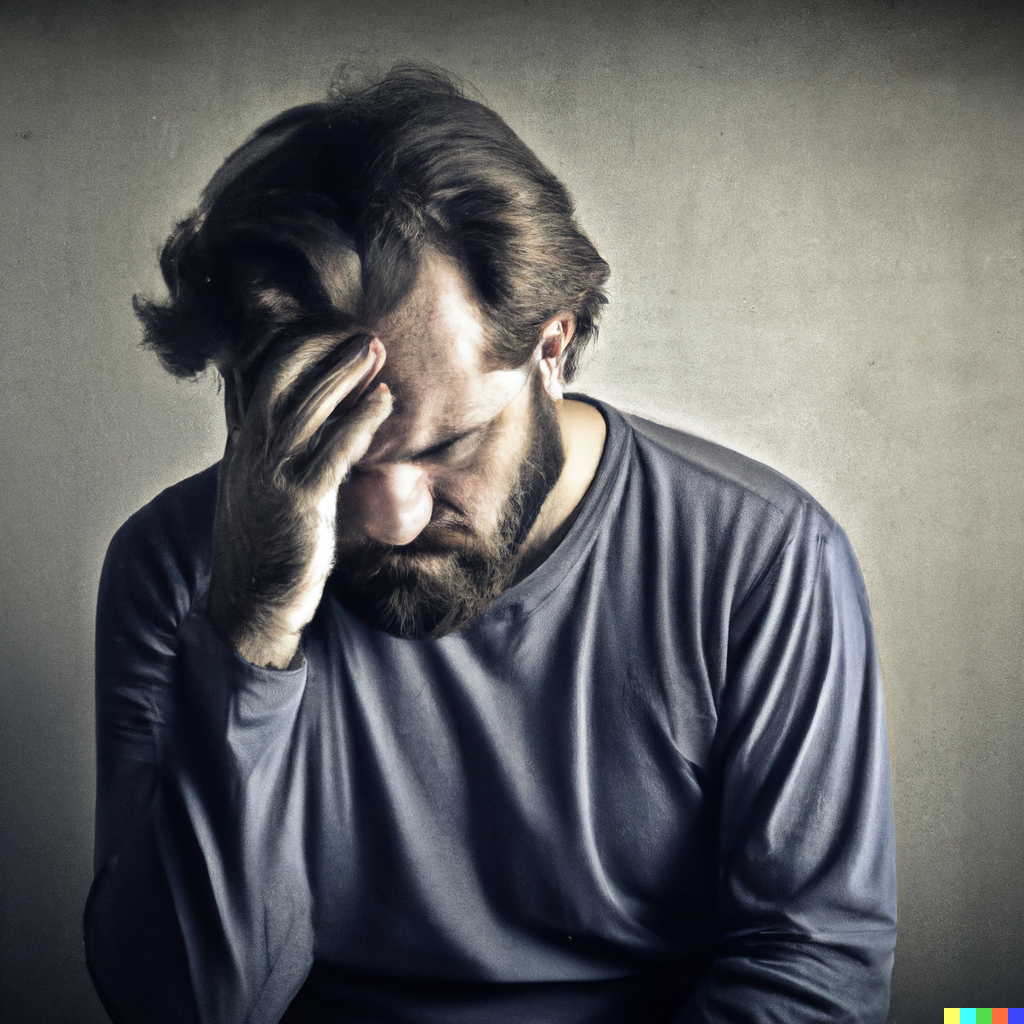 |
| Dream Studio | 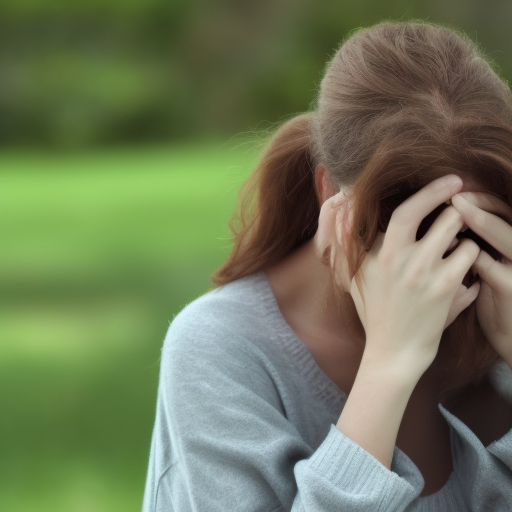 |
| Dream Studio | 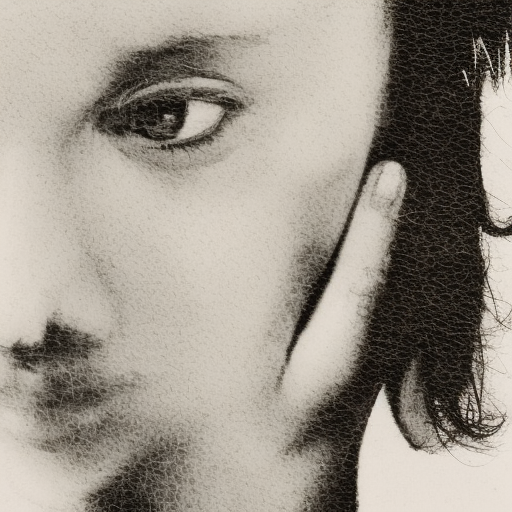 |
| Dream Studio | 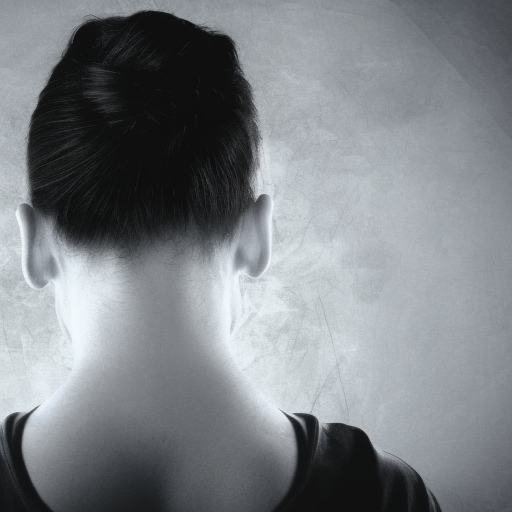 |
