# Supplementary material for: Comparing Images of Depression in Mass Media and AI-Generated Pictures: Mixed Methods Study
Source: JMIR Hum Factors. 2026 Apr 14;13:e81230. doi: 10.2196/81230 (PMC13094379; doi:10.2196/81230)
Supplement: Multimedia Appendix 4 [file humanfactors-v13-e81230-s004.docx]

**Supplementary material 4**

**Surveys**

Screening: Gender, Age, Region

**Q0. Select which of the following conditions you have been diagnosed with by a doctor:**

1. Migraines

2. Obsessive-Compulsive Disorder (OCD)

3. Attention Deficit Hyperactivity Disorder (ADHD)

4. Panic Attacks

5. Anxiety

6. Depression

7. None of the above

***TEST GROUP***

Below, you will see two sets of images. The first set consists of images used by media outlets to illustrate news stories about depression. The second set contains images generated by artificial intelligence after being asked to illustrate the word "depression." Once you have looked at the images, we will ask you some questions so you can evaluate them.

- *Images used by the media (displayed randomly)*
- *Images generated by artificial intelligence (displayed randomly)*

**Q1. In your opinion, which 5 images are most appropriate to represent depression?** *(Display images on a single screen, allowing users to click on them)*

**Q2. In your opinion, which 5 images most realistically represent depression?** *(Display images on a single screen, allowing users to click on them)*

**Q3. In your opinion, which 5 images are the most stereotypical?** *(Display images on a single screen, allowing users to click on them)*

**Q4. In your opinion, which 5 images best present the relationship between gender and depression?** *(Display images on a single screen, allowing users to click on them)*

**Q5. In your opinion, which 5 images are the most inclusive?** *(Display images on a single screen, allowing users to click on them)*

**Q6. In your opinion, which 5 images most strongly reinforce stigmas of marginalization or social exclusion?** *(Display images on a single screen, allowing users to click on them)*

**Q7. In your opinion, which 5 images are most likely to negatively affect people with depression that view them?** *(Display images on a single screen, allowing users to click on them)*

**Q8. How do you think the images could be improved? (Recommendations)**

*(open text box)*

**Q9. Select the statements that can help improve how depression is depicted.** *(Multiple choice option, more than one option can be ticked)*

- Show people with depression going about their normal lives: with family, friends, at work, having fun, doing everyday activities, etc.
- Show more diversity in the images (different skin tones, gender diversity, age, disability diversity, different body types, etc.)
- Show that help is available, that there are options for therapy, support, and counselling.
- Consider that depression has many degrees and that a single image cannot illustrate everything that depression represents.
- Consult with mental health specialists when choosing images.
- Other: _______

**Q10. Who do you think is the main responsible for the impact that images generated using artificial intelligence may have?** *(Multiple choice option, more than one option can be ticked)*

- The communicator who uses the image
- The managers of the companies that own the media outlets
- The managers of the companies that develop artificial intelligence
- The technical staff who design artificial intelligence at the companies that develop it
- Governments
- Everyone has a similar responsibility
- Other: ______

**Q11. What aesthetic factors of the images caught your attention the most? (***Multiple-choice option, more than one option can be ticked)*

- Colours used
- Presence/absence of text
- Other: ________

Below, you will see images recommended by three mental health organizations (two Spanish and one British) to illustrate depression and mental health. These organizations advocate for the use of this type of images over more stereotypical ones. After viewing these images, we will ask you a few questions so you can evaluate them.

- Recommendations from organization 1
- Recommendations from organization 2
- Recommendations from organization 3

**Q12. What is your opinion of the following images?** Rate them from 0 to 5, where 0 means the images are very unsuitable for representing depression, and 5 means the images are very suitable for representing depression. (*Single response option, only one box can be checked)*

*Scale*

*0 ___ 1 ___ 2 ___ 3 ___ 4 ___ 5*

**Q13. What aspects of these images do you think are appropriate for illustrating mental health or depression?** *(Open text box)*

**Q14. How do you think these images could be improved? (Recommendations)** *(Open text box)*

***CONTROL GROUP***

Below, you will see 30 images used to illustrate depression. After viewing the images, we will ask you a few questions so you can rate them.

- *Images by the media and AI-generated (displayed randomly)*

**Q1. In your opinion, which 5 images are most appropriate to represent depression?** *(Display images on a single screen, allowing users to click on them)*

**Q2. In your opinion, which 5 images most realistically represent depression?** *(Display images on a single screen, allowing users to click on them)*

**Q3. In your opinion, which 5 images are the most stereotypical?** *(Display images on a single screen, allowing users to click on them)*

**Q4. In your opinion, which 5 images best present the relationship between gender and depression?** *(Display images on a single screen, allowing users to click on them)*

**Q5. In your opinion, which 5 images are the most inclusive?** *(Display images on a single screen, allowing users to click on them)*

**Q6. In your opinion, which 5 images most strongly reinforce stigmas of marginalization or social exclusion?** *(Display images on a single screen, allowing users to click on them)*

**Q7. In your opinion, which 5 images are most likely to negatively affect people with depression that view them?** *(Display images on a single screen, allowing users to click on them)*

**Q8. How do you think the images could be improved? (Recommendations)**

*(open text box)*

**Q9. Select the statements that can help improve how depression is depicted.** *(Multiple choice option, more than one option can be ticked)*

- Show people with depression going about their normal lives: with family, friends, at work, having fun, doing everyday activities, etc.
- Show more diversity in the images (different skin tones, gender diversity, age, disability diversity, different body types, etc.)
- Show that help is available, that there are options for therapy, support, and counselling.
- Consider that depression has many degrees and that a single image cannot illustrate everything that depression represents.
- Consult with mental health specialists when choosing images.
- Other: _______

*[From this point, it is not possible to go back and look at the images]*

**Q10. Some of the images you just looked at were generated using artificial intelligence (AI). Who do you think is primarily responsible for the impact that AI-generated images can have?** *(Multiple choice option, more than one option can be ticked)*

- The communicator who uses the image
- The managers of the companies that own the media outlets
- The managers of the companies that develop artificial intelligence
- The technical staff who design artificial intelligence at the companies that develop it
- Governments
- Everyone has a similar responsibility
- Other: ______

**Q11. What aesthetic factors of the images caught your attention the most? (***Multiple-choice option, more than one option can be ticked)*

- Colours used
- Presence/absence of text
- Other: ________

Below, you will see images recommended by three mental health organizations (two Spanish and one British) to illustrate depression and mental health. These organizations advocate for the use of this type of images over more stereotypical ones. After viewing these images, we will ask you a few questions so you can evaluate them.

- Recommendations from organization 1
- Recommendations from organization 2
- Recommendations from organization 3

**Q12. What is your opinion of the following images?** Rate them from 0 to 5, where 0 means the images are very unsuitable for representing depression, and 5 means the images are very suitable for representing depression. (*Single response option, only one box can be checked)*

*Scale*

*0 ___ 1 ___ 2 ___ 3 ___ 4 ___ 5*

**Q13. What aspects of these images do you think are appropriate for illustrating mental health or depression?** *(Open text box)*

**Q14. How do you think these images could be improved? (Recommendations)** *(Open text box)*
